# A novel tool for the unbiased characterization of epithelial monolayer development in culture

**DOI:** 10.1091/mbc.E22-04-0121

**Published:** 2023-03-07

**Authors:** Nicole S. Dawney, Christian Cammarota, Qingyuan Jia, Alicia Shipley, Joseph A. Glichowski, Muskaan Vasandani, Tara M. Finegan, Dan T. Bergstralh

**Affiliations:** a Department of Biology, University of Rochester, Rochester, NY 14627; b Department of Physics & Astronomy, University of Rochester, Rochester, NY 14627; c Department of Biomedical Genetics, University of Rochester Medical Center, Rochester, NY 14627; University of Queensland

## Abstract

The function of an epithelial tissue is intertwined with its architecture. Epithelial tissues are often described as pseudo–two-dimensional, but this view may be partly attributed to experimental bias: many model epithelia, including cultured cell lines, are easiest to image from the “top-down.” We measured the three-dimensional architecture of epithelial cells in culture and found that it varies dramatically across cultured regions, presenting a challenge for reproducibility and cross-study comparisons. We therefore developed a novel tool (Automated Layer Analysis, “ALAn”) to characterize architecture in an unbiased manner. Using ALAn, we find that cultured epithelial cells can organize into four distinct architectures and that architecture correlates with cell density. Cells exhibit distinct biological properties in each architecture. Organization in the apical-basal axis is determined early in monolayer development by substrate availability, while disorganization in the apical-basal axis arises from an inability to form substrate connections. Our work highlights the need to carefully control for three-dimensional architecture when using cell culture as a model system for epithelial cell biology and introduces a novel tool, built on a set of rules that can be widely applied to epithelial cell culture.

## INTRODUCTION

Epithelial tissues form structural and protective barriers between body compartments in animals. Epithelia are composed of mostly identical cells that share an apical-basal polarity and are mechanically linked via lateral cell–cell junctions to form confluent sheets (Buckley and St Johnston, 2022). Simple epithelia are the most common architecture and are one-cell-thick monolayer sheets. This architecture facilitates specialized functions such as absorption, secretion, and the formation of compartments with distinct ionic composition (Fristrom, 1988). Disruption of epithelial architecture, particularly in the apical-basal axis, is a hallmark of disease. These include solid carcinomas, which are the most common type of cancer (McCaffrey and Macara, 2011), and rare diseases such as congenital tufting enteropathy, which is characterized by gaps between cells and a resultant reduced ability to absorb nutrients (Das and Sivagnanam, 2020).

Given that epithelia are the most abundant tissue type in the animal body, and that they are the origin of most adult cancers, attention has long been given to mechanisms that govern the arrangement of their component cells (Lemke and Nelson, 2021). These studies have generally focused on the tissue “plane,” which is to say across the tissue rather than with respect to its depth (Thompson, 1917; Kikuchi, 1956; Staple *et al.*, 2010). However, cells are three-dimensional (3D) objects. Cell shapes and their packing along the apical-basal axis, perpendicular to the tissue plane, are far less studied. This is in part because it has been difficult to penetrate into the depth of a tissue due to light scattering, and hence conventional one-photon microscopy techniques have meant that the lateral and basal surfaces have been difficult to resolve (Suan *et al.*, 2013). Furthermore, computational limitations have meant that assuming epithelia as pseudo–two-dimensional structures has been convenient for image analysis and modeling purposes.

Technological advances that facilitate rapid multidimensional confocal imaging and high-content image analysis allow us to tackle the question of epithelial monolayer architecture in 3D. One of the premier models for this work is the widely used Madin–Darby canine kidney (MDCK) epithelial cell line, which can form a continuous sheet of polarized cells when grown on permeable filter inserts or a collagen substrate (Cereijido *et al.*, 1978; Simmons, 1982; Dukes *et al.*, 2011). One drawback to this cultured system is the difficulty in making comparisons across studies. It has long been recognized that MDCK cell organization and shape can be variable between clones and from lab to lab (Gaush *et al.*, 1966; Husted *et al.*, 1986; Dukes *et al.*, 2011). Furthermore, small differences in culture conditions (between labs or even individuals) might impact architecture in 3D. We found that organization can vary even within a single culture well (1 cm × 1 cm).

Some epithelial tissues, sometimes referred to as secondary epithelia, are formed from a mesenchymal-to-epithelial transition (MET). In this process, single migratory cells come together and transform their identity to form epithelial sheets. Developmental examples are vertebrate somitogenesis and midgut morphogenesis in *Drosophila* (Yarnitzky and Volk, 1995; Pourquié, 2001)*.* MET is relatively not well characterized in comparison to its opposite, epithelial-to-mesenchymal transition (EMT). In particular, the physical parameters that control epithelial layer development from single cells remain unexplored (Bernardi and Strobl-Mazzulla, 2021; Sheng, 2021). We sought to characterize the dynamics of epithelial layer formation in 3D using the passaging of cultured mammalian epithelial cells, which could be considered an MET process.

We set out to characterize epithelial monolayer architecture and development in an unbiased manner. To do so, we developed a novel tool, which we call Automated Layer Analysis (ALAn), filling a gap in current analysis technology to determine 3D layer architecture of cells in culture.

## RESULTS

### The architecture of cultured MDCK cell layers can vary drastically across small regions

**FIGURE 1:**
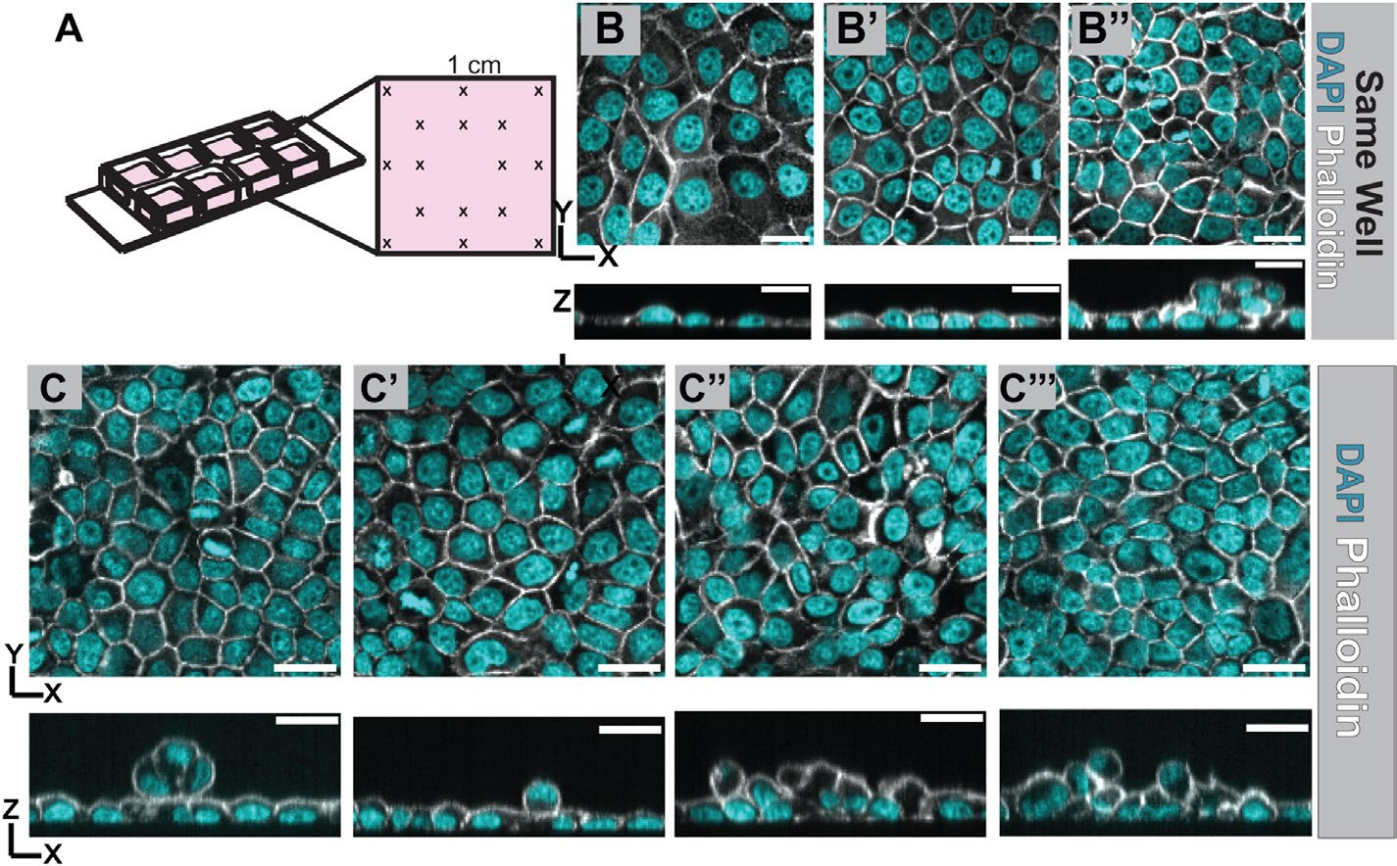
The 3D architecture of cultured MDCK cell layers can vary drastically across small regions. (A) Schematic of the collagen-coated chamber slide used to grow cell culture monolayers. Each well is 1 cm × 1 cm, and the same 16 positions in each well are imaged to minimize user bias. (B) Regions within one well can vary drastically, from flat, sparse cells (B) to more regular architecture with lateral surfaces being more prominent (B′). Cells can also look regular in the *XY* plane but have multilayering when looking in the *XZ* dimension (B′′). (C) Using the same culture conditions, we observe a variety of multilayering topologies that cannot be distinguished by looking at the *XZ* axis only. *XY* views are generated by making a Sum projection through many *Z* planes encompassing the depth of the layer. *XZ* views are generated by reconstructing confocal z slices through one *Y* plane. Scale bars = 20 µm.

We are interested in how monolayer tissue architectures form and what factors influence their structure. While we understand much about the organization of epithelial cells in the apical junctional plane, we know less about cell architecture in the third dimension. We thus set out to characterize the transition of single, dissociated cells into an organized epithelial monolayer while following their shapes in 3D. We plated dissociated MDCK cells in collagen-coated chamber slides with wells of area 1 cm^2^ (Figure 1A). We then performed time-course experiments whereby we fixed and imaged cells following initial plating and through the process of layer formation. Strikingly, we found that within a single 1 cm^2^ culture area, cultured over the same time and under the same conditions, MDCK monolayer architecture can vary dramatically in 3D (Figure 1, B, B′, and B′′). Critically, we found that cultured tissues that appear ordered in the tissue plane can show gross, variable disorganization in the apical-basal plane (Figure 1, B and C). We therefore concluded that it is not sufficient to assume that a cultured tissue is a monolayer without imaging the full depth of the layer. We also noted that the height and 3D shape of cells can vary between cultured monolayers and noticed that this tended to be correlated with the time since cell plating. We therefore set out to quantitatively characterize cell shape and tissue architecture in cultured epithelial layers and investigate how this relates to biological properties of cells, such as intracellular polarity, junctional composition, and material properties.

### ALAn: a novel tool to classify cultured epithelial layer architecture

**FIGURE 2:**
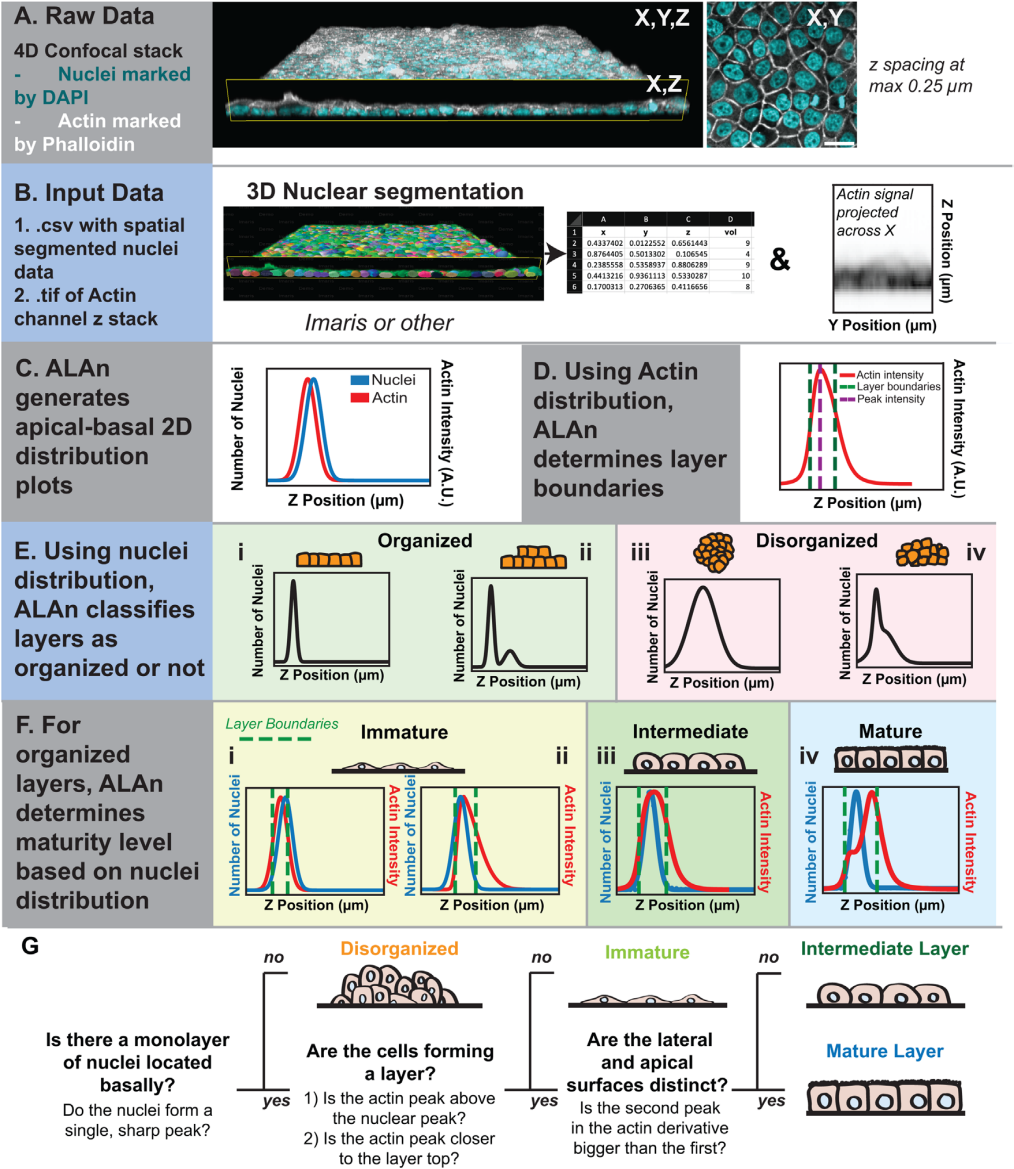
ALAn: a novel tool to classify cultured epithelial layer architecture. (A–F) Stages of epithelial architecture analysis using ALAn. Graphs show representative distributions of actin and nuclei used to classify layers. (E) If the nuclei form a single sharp peak (i) or two separate peaks (ii), the layer is classified as organized. If the nuclei form a single wide peak spanning multiple Z positions (iii) or two overlapping peaks (iv), the layer is classified as Disorganized. (F) Example plots represent how Immature layers are defined; the morphology of cells results in an actin peak below the nuclear peak (i) and/or the peak of the actin plot is closer to the bottom of the layer rather than the top. (iii) Intermediate layers have monopeaked actin intensity plots. (iv) Mature monolayers display a two-step growth to the peak intensity reflected in the actin plot. (G) Flowchart providing a visual explanation of how ALAn determines layer architecture.

To systematically and quantitatively assess both the nature and underlying sources of tissue architecture variability, we needed to be able to classify epithelial architectures in an unbiased manner and to do this for many cells within a culture and at many time points during epithelial development. This prompted us to develop a novel tool, which we call ALAn. ALAn is based on the idea that layer architecture could be described and categorized based on 1) the positions of cell nuclei, which give information about the number and positions of individual cells; and 2) the cellular actin cytoskeletons, which give information about cell–cell junctions and cell apical and basal domains. ALAn is a Python script that takes two inputs: 1) a .csv file that contains the positional information of nuclei derived from image segmentation; and 2) the corresponding 3D .tif image file containing the spatial distribution of actin in the cultured tissue layer (Figure 2A). These inputs are obtained by imaging monolayers grown on collagen-coated chamber slides immunostained with DAPI (to visualize 4′,6-diamidino-2-phenylindole [DNA]) and phalloidin (to visualize F-actin). To ensure that the full depth of the tissue layer is imaged, z-stacks are set up to encompass all intracellular actin signal at a z-resolution of at least 0.5 µm. The 3D positional information for each nucleus is obtained using a segmentation tool based on the DAPI stain; we used the convenient “Cell Segmentation” tool in Imaris (Andor) (Figure 2B). The centroid of each nucleus, the relative position of these centroids in relation to the top and bottom of the layer, and the absolute nuclei volumes are exported following segmentation. No further parameters about the nuclear shapes are necessary to classify the architecture of layers.

ALAn first generates a spatial distribution of nuclei along the apical-basal (*z*) axis based on the segmented nuclear positional information (Figure 2C). ALAn removes any objects that are too small to be nuclei. As segmented nuclear volume can vary with image quality, the cutoff for nuclear volume is set on a per-image basis; any nuclear volume 1.5 SDs below the average volume or lower is excluded (Supplemental Figure S1A). We did not use an upper cutoff, because very large nuclei are occasionally observed in MDCK cell culture (Supplemental Figure S1A′). To characterize whether nuclei are arranged in a single monolayer or are multilayered, the spatial distribution of nuclei in the *z* dimension is then approximated by a histogram with 1 µm bin spacing (Supplemental Figure S1B).

The upper and lower bounds to any cellular layer are determined using actin intensity (Figure 2D). As cell size and height vary across a region of interest and with cell density, the peak of the actin intensity plot does not correspond to the top of the layer. The bottom of the layer corresponds to the first plane in the image where the actin intensity increases more than 20–50% of the peak intensity, dependent on cell density. Likewise, the top of the layer is set to the last plane with an actin intensity above 60–80% of the peak (Supplemental Figure S1C). To verify ALAn’s accuracy in layer detection and determination, we manually determined the focal planes corresponding to the top and bottom of the layer for a subset of images and used these determinations to calibrate the tool (Supplemental Figure S1D).

Using the nuclear distribution and the layer bounds, ALAn then checks to see whether the cells are ordered or disordered with respect to layering (Figure 2E). To do so, ALAn determines whether the spatial distribution of nuclei in *z* is best fitted by one or two Gaussian functions. If the two-Gaussian-function fit is significantly different from the one-Gaussian fit, the distribution is classed as having two peaks (Supplemental Figure S1E). Nuclei in a single organized layer will be fitted by a single Gaussian curve (Figure 2Ei). If a single organized layer is present with some cells on top of this layer, the nuclei spatial distribution function will be described by a two-Gaussian distribution with distinct peaks (Figure 2Eii). If the layer is Disorganized, the nuclei will either be fitted by a single, wide Gaussian function (Figure 2Eiii; Supplemental Figure S1F) or be best fitted by a two-Gaussian distribution, but with overlapping peaks (Figure 2Eiv).

Organized monolayers are then further subcategorized by ALAn into Immature, Intermediate, or Mature, using the relationship between the actin intensity and nuclear distribution (Figure 2F). The tool projects the spatial distribution of the actin signal onto the *z*-axis by summing the intensity through the *x* and *y* coordinates. Immature layers are characterized by poorly defined lateral surfaces (Figure 2F). Therefore, in these layers the peak nuclear distribution is located at, or occasionally above, the peak actin intensity (Figure 2F, i and ii). Both Intermediate and Mature layers demonstrate defined lateral surfaces; the cells in an Intermediate layer have curved apical surfaces (Figure 2Fiii), whereas the cells of Mature layers have flat apices (Figure 2Fiv). This results in characteristic differences in the plots of the actin intensity versus *z* position. The actin curve with respect to *z* position for Mature layers results in a pronounced left shoulder (Figure 2Fiv and Supplemental Figure S1G). The shoulder represents lateral actin. The actin signal distribution increases after the shoulder which corresponds to the actin-enriched, flat apical surface. ALAn uses the derivative of the actin intensity plot to detect this shoulder. If the derivative is two-peaked, the ratio of the right peak to the left peak is used. A ratio of greater than 1 is classed as a Mature layer (Supplemental Figure S1G′). In contrast, actin intensity plots for Intermediate layers are monopeaked (Figure 2Fiii and Supplemental Figure S1H). Either the Intermediate layers therefore have a single peak in the actin derivative or the ratio of the peaks is less than 1 (Supplemental Figure S1H′). ALAn also outputs the number of cells both within and above an organized layer, a parameter that we further tested by manual counts (Supplemental Figure S1I). We developed a simple flowchart to provide a visual explanation of how ALAn determines layer organization based on the rules outlined above (Figure 2G).

**FIGURE 3:**
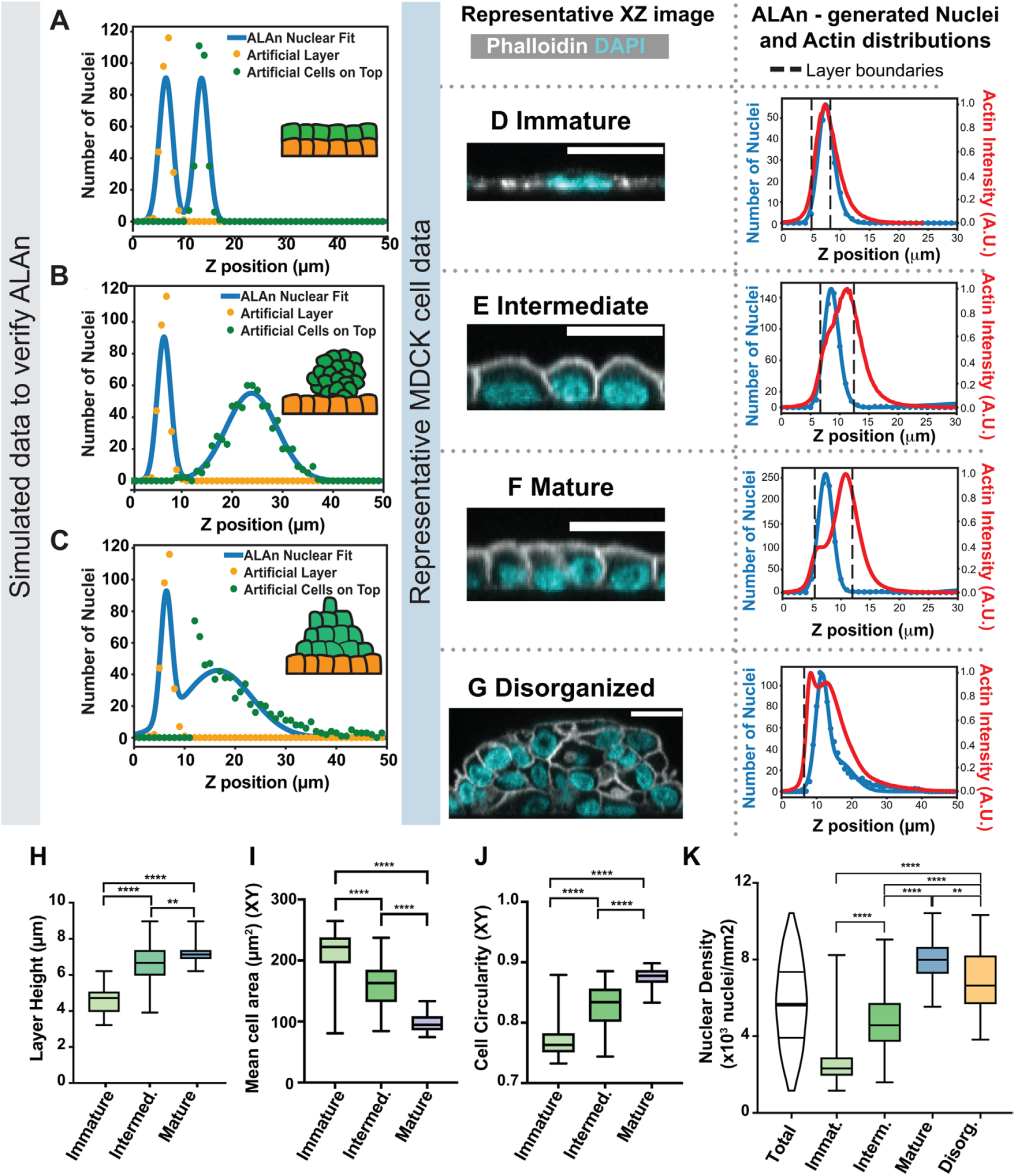
ALAn categorizes epithelial layers into four distinct architectures. (A–C) Simulated layers were made to test ALAn that consisted of either a perfect monolayer (A), a ball of cells on top of an organized layer (B) or a “pyramid” of cells on top of an organized layer (C). As there is a monolayer underneath, ALAn classifies all three as organized. (D–G) Table summarizing the layer architecture types and representative images and plots characteristic of each category. (D) Immature: Component cells are relatively flat, producing a plot with nuclear centroids above the actin peak. Scale bars = 20 µm. (E) Intermediate: Component cell have domed apices, shifting the nuclear peak below the actin peak. (F) Mature: Component cells are regular, with flat apices. A “shoulder” in the actin intensity plot appears. (G) Disorganized: Nuclei are broadly distributed, as revealed in the histogram of nuclear z-position. (H) Layers increase in height with maturity, with Immature layers being the shortest (average of ∼4.5 µm) and Mature layers the tallest (average of ∼7 µm). *P* values left to right: *p* < 0.0001, *p* < 0.0001, *p* = 0.0034. (I) Mean cell area decreases as layers mature, with cells in Immature layers having an area of ∼220 µm^2^, decreasing to ∼91 µm^2^ for cells in Mature layers. *P* values left to right: *p* < 0.0001, *p* < 0.0001, *p* < 0.0001. (J) Cells become more regular as layers mature, shown by an increase in circularity from ∼0.76 for Immature cells up to ∼0.88 for Mature cells. *P* values left to right: *p* < 0.0001, *p* < 0.0001, *p* < 0.0001. (K) Layer architectures correspond to different cell densities. *P* values left to right: *p* < 0.0001, *p* < 0.0001, *p* < 0.0001, *p* < 0.0001, *p* = 0.0032. All statistics unpaired, two-tailed Student’s *t* test. (H, I, J, and K) *n* = 48 Immature, 141 Intermediate, 55 Mature, 109 Disorganized.

To test whether ALAn can identify organized layers in all circumstances, we generated artificial “extreme” examples of organized layers. These consist of 1) An ordered double layer, simulated by generating a spatial distribution function of nuclei composed of two distinct Gaussian distributions with small SDs (Figure 3A). 2) A large disorderly clump of cells sitting above an underlying organized layer; simulated by a nuclear spatial distribution composed of a sharp basal nuclear peak and a tall, broad Gaussian curve peaking to the right of (above) the first (Figure 3B). 3) A pyramid of cells sitting atop an organized layer; simulated by a nuclear distribution composed of a sharp basal nuclear peak and a decaying exponential curve (Figure 3C). ALAn correctly identified all these “extreme” examples as organized, demonstrating that our rules can classify organized layers no matter the morphology of extralayer cells.

In summary, ALAn allows users to plot the distributions of actin and DNA for any given image in the apical-basal axis. We determined a set of rules that ALAn uses to classify tissue architecture. Cells in the organized architectures have three distinct morphologies; cells in Immature layers are flat (Figure 3D, column 1), resulting in nuclear centroids that are positioned at or above the actin intensity peak (Figure 3D, column 2). Intermediate layer cells are characterized by domed apices (Figure 3E), consistent with the cobblestone morphology long described in cultured epithelial models (for example, Behrens *et al.*, 1985; Mostov and Deitcher, 1986). In contrast, Mature layers are distinguished by flat apical surfaces, resulting in an intense apical band of actin (Figure 3F). Disorganized layers are distinct in that there is no discernible monolayer (Figure 3G).

### The four architectures of cultured epithelial cells exhibit distinct biological and material properties

Having identified four architectures that MDCK cells can form, we used ALAn to further define the characteristics of each architecture. We found that organized layers increase in height as they transition through their three stages; Immature layers have mean layer heights of 4.6 µm, while Intermediate and Mature layers are generally taller, with mean heights of 6.5 and 6.9 µm, respectively (Figure 3H). In addition to classifying the input layer, ALAn allows users to determine the in-plane shape and size of cells. Cells in Intermediate and Mature layers have smaller cross-sectional areas and more regular in-plane shapes than cells in the Immature class (Figure 3, I and J). Finally, across our data set, the three organized layer architectures are separated by nuclear density (total number of nuclei per culture area), with some overlap between them (Figure 3K).

**FIGURE 4:**
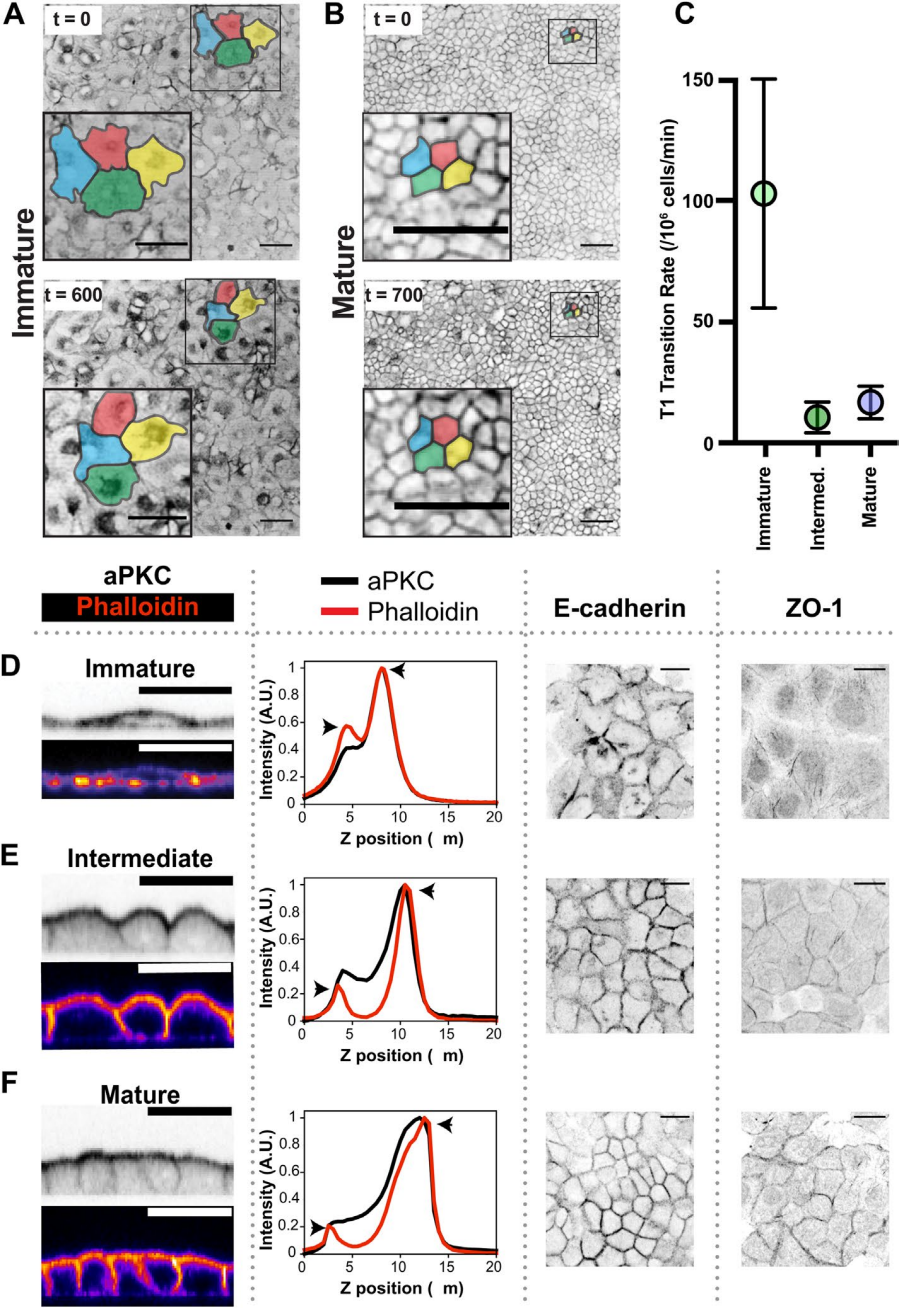
The material and biological properties of cells are distinct in different layer architectures. (A, B) Fluidity and cell rearrangements decrease between immature and mature organized architectures. Frequent T1 transitions are observed in Immature layers, with fewer transitions being observed in Intermediate layers, and little to no transitions being observed in Mature layers. (C) Quantification of T1 transitions in Immature, Intermediate, and Mature movies (Supplemental Movies 1, 2, and 3). Scale bars = 50 µm. (D) Immature; (E) Intermediate, (F) Mature. aPKC shows apical localization in all three layer types (columns 1 and 2), and E-cadherin is observed at all cell–cell borders (column 3). Cortical actin shows a twofold increase at the apical surface in Immature layers and is more asymmetric in the Intermediate and Mature layers (column 2, indicated by arrows). ZO-1 immunoreactivity is not observed in Immature layers and develops as layers mature (column 4). Scale bars = 20 µm.

Previous work using cultured epithelial cells suggests that cells at lower densities move faster and farther in the plane of the tissue than their densely packed counterparts (Angelini *et al.*, 2011; Devany *et al.*, 2021). Given the density distinctions between Immature, Intermediate, and Mature layers, we probed whetherthe dynamic behaviors of cells in each of the layer architectures differed. To test the movement of cells displaying the different architectures, we performed live imaging on layers stained with the lipid dye CellMask (ThermoFisher) (Figure 4, A and B). For each of the organized layer architectures, we measured the rate at which cells within the layer move by analyzing the membrane intensity correlation between successive time points (Supplemental Figure S2A). This analysis revealed that the correlation half-life for the Immature movie is 287 min, while the Intermediate and Mature movies have half-lives of 424 and 754 min, respectively (Supplemental Figure S2, B and B′). Thus, the membrane intensity in Immature layers decorrelates three times faster than that of Mature layers. Cell rearrangements are a hallmark of tissue fluidity; the frequency with which cells exchange neighbors is negatively correlated to the development of cell–cell and cell–substrate adhesions (Bi *et al.*, 2015; Garcia *et al.*, 2015; Merkel and Manning, 2017). We determined the rate of neighbor exchanges in the three organized layer architectures and found that cell–cell junctions rearrange much more frequently in Immature layers (∼103 transitions per 10^6^ cells per minute) in comparison to Intermediate and Mature layers (∼10 and 16 transitions per 10^6^ cells per minute, respectively) (Figure 4, A–C). These data suggest that cells within MDCK monolayers undergo a transition from fluid-like to glass-like in the tissue plane during the Immature to Intermediate transition correlating with a density increase, further suggesting that this change in the viscoelastic properties of the tissue layer is a density-dependent transition.

**Figure:** Movie S1 **Immature Layer Fluidity** A monolayer which appears to be Immature in architecture undergoes frequent neighbor exchanges. Movie taken at 10 minutes / frame shown at 10 frames / second.

**Figure:** Movie S2 **Intermediate Layer Fluidity** A monolayer which appears to be Intermediate in architecture shows a significant glasslike nature with some neighbor exchanges. Movie taken at 10 minutes / frame shown at 10 frames / second.

**Figure:** Movie S3 **Mature Layer Fluidity** A monolayer which appears to be Mature in architecture has transitioned from fluid to glasslike with little to no neighbor exchanges. Movie taken at 10 minutes / frame shown at 10 frames / second.

### Transition between the three architectures is accompanied by changes in the localization of apical and junctional proteins

In addition to shape and dynamic distinctions, we looked for molecular differences between the epithelial layer architectures. We characterized the localization of the apical polarity marker aPKC (Wang and Margolis, 2007) and the junctional components E-cadherin (adherens junctions) and ZO-1 (tight junctions) in each architecture (Martin-Belmonte and Perez-Moreno, 2012). All three of the organized architectures exhibit predominantly apical localization of aPKC (Figure 4, D–F), consistent with previous work demonstrating that cell–substrate adhesion alone can initiate apical surface identity (Vega-Salas *et al.*, 1987). In contrast, we found that aPKC in Disorganized architectures localizes to cell–cell borders (Supplemental Figure S3A), suggesting that polarity is absent in Disorganized architectures. Furthermore, while the adherens junction component E-cadherin is localized to lateral cell–cell junctions in all three organized layer types, strong junctional staining of the tight junction marker ZO-1 is distinct to Mature layers (Figure 4, D–F, column 4; Supplemental Figure S3B). A final difference between the three organized architectures is the intensity of the apical actin signal. Apical actin is characteristic of an apical brush border, a structure that maximizes absorption in the kidney tubule epithelial cells. Apical actin enrichment relative to lateral actin is strongest in Mature layers and less pronounced in Immature layers (Figure 4, D–F, column 2). These results show that the different layer architectures have distinct molecular polarity profiles.

### ALAn is versatile

Having designed and verified ALAn for assessing fixed MDCK cell monolayers, we next tested whether the tool can be used to classify cell layers imaged live. We cultured an MDCK monolayer and used vital dyes to test the applicability of ALAn on live samples. Layers plated at different seeding densities were left for 24 h and imaged live using Hoechst staining to identify and segment nuclei and SiR-actin to visualize actin. The SiR-actin profile generally reflects the layer architecture profiles distinguished from phalloidin staining (Supplemental Figure S4A). ALAn may be used to classify epithelial tissues imaged live as long as the actin staining is homogeneous across the imaged tissue.

**FIGURE 5:**
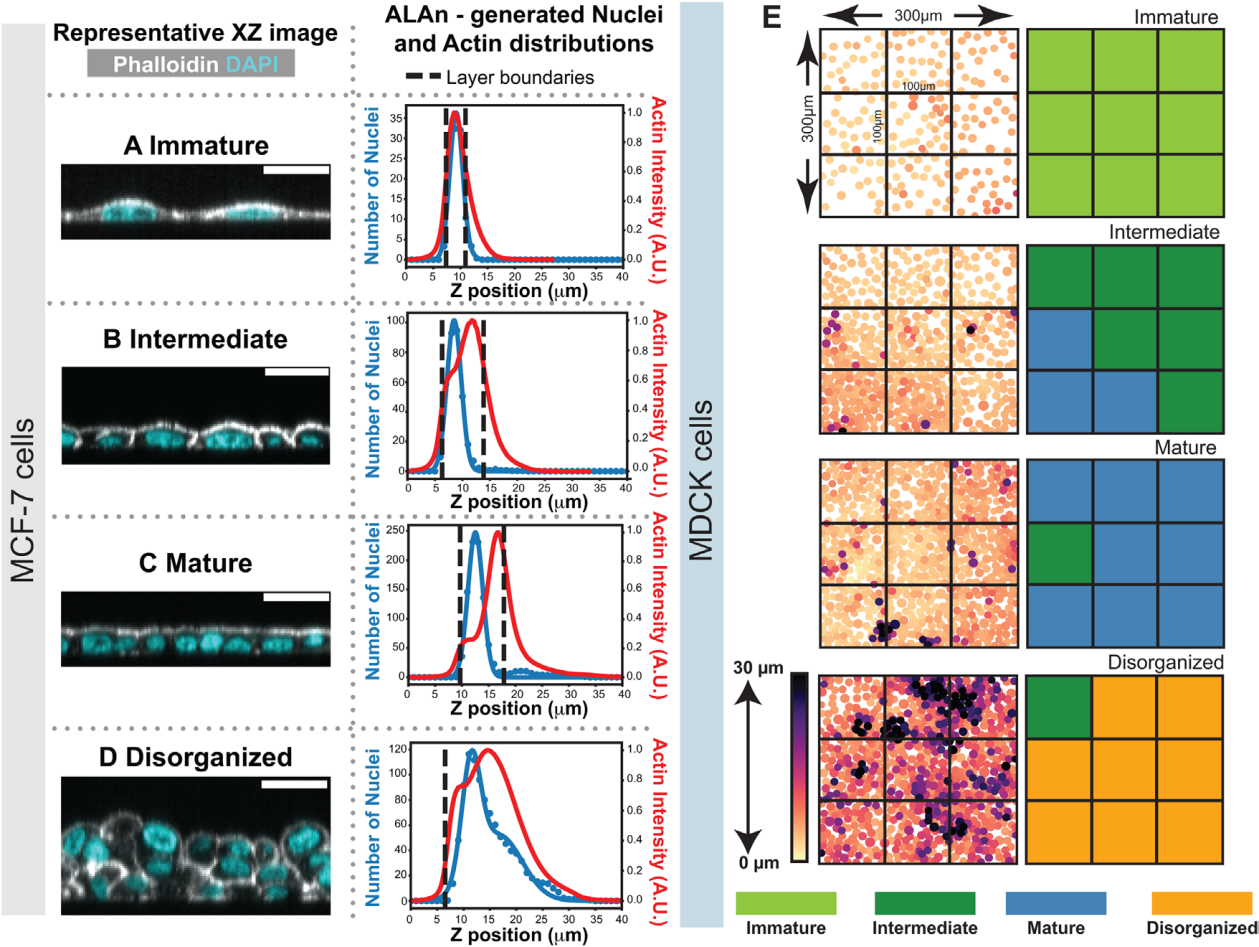
Versatility of ALAn across cell types and spatial scales. (A–D) Example *XZ* images and plots for each layer type found in MCF-7 cell cultures. (E) ALAn can subclassify images accurately, with the majority of subclassifications defining overall classification. Color dictates cell centroid position with respect to the layer bottom.

We next asked whether ALAn can be used to classify tissue architecture in other epithelial cell culture lines representing distinct tissues of origin. ALAn readily classified MCF-7 human breast cancer epithelial cells, revealing the same four architectures as determined for MDCK (Figure 5, A–D). We found that the topological layer characteristics of the MCF-7 cells were similar to those of MDCK cells (Supplemental Figure S4, B–E).

In contrast, Caco-2 human colon cancer cells exhibited different patterns of cellular actin and thus classification requires adjustments to ALAn. Caco-2 monolayers exhibited a prominent basal actin peak not observed in MDCK or MCF-7 monolayers (Supplemental Figure S4, F–I). As Caco-2 cells transitioned to more mature states, they also exhibited a clear apical actin peak. This allowed us to identify the patterns of actin in Caco-2 layers at different cell densities and develop Caco-2–specific layer classifications (Supplemental Figure S4, F–I).

Because ALAn uses two simple and universal parameters (distributions of actin and nuclei) to determine epithelial layer architecture, our data suggest that ALAn can be widely used to characterize the epithelial cell culture architectures of different cell types and origins. We expect the rules based on nuclei distribution to be widely applicable to all cells that form layers. We have found that actin distribution in the apical-basal axis can vary between different epithelial culture types. Minor adjustments to the specific cutoffs within ALAn are required for different epithelial culture types. However, our work provides a readily modified architecture analysis tool for confluent cell layers.

### Using ALAn at different spatial scales to determine the variability in architectures across cultured regions

To test whether ALAn can be used to classify the architectures of subregions within an epithelial monolayer, we returned to MDCK cells and measured the cell density within differently sized square regions ranging from 25 × 25 µm to 300 × 300 µm. In agreement with previous work, we found that the density within small subregions is largely representative of a larger 300 × 300 µm region, although the variability in measured cell density increases as the subregion size decreases (Supplemental Figure S5A) (Devany *et al.*, 2022). Few regions in the 25 × 25 µm boxes display a density of 0 cells as regions this small sometimes do not contain nuclear centroids (Supplemental Figure S5B). We plotted the cell density for 25 × 25 µm and 100 × 100 µm regions against the measured density for our full microscope field of view, 300 × 300 µm. We found that the cell densities for the smaller regions correlate well to that of the full image, with the slopes of the lines of best fit being 1.054 and 1.014 for the 25 and 100 µm boxes, respectively (Supplemental Figure S5C).

Having verified the cell density of the smaller regions to be representative, we next split our 300 µm × 300 µm images into nine 100 µm ×100 µm regions to run through ALAn. Strikingly, layer architecture varies between the subregions (Figure 5E). However, the ALAn layer classification of the full 300 × 300 µm field of view matches the mode classification of the nine subregions (Figure 5F). It is important to note that while the regions we tested were accurately classified, smaller region areas may lead to decreased classification accuracy as ALAn determines layer architecture based on averages of actin and nuclear signals.

### Architectures transition as layers densify

**FIGURE 6:**
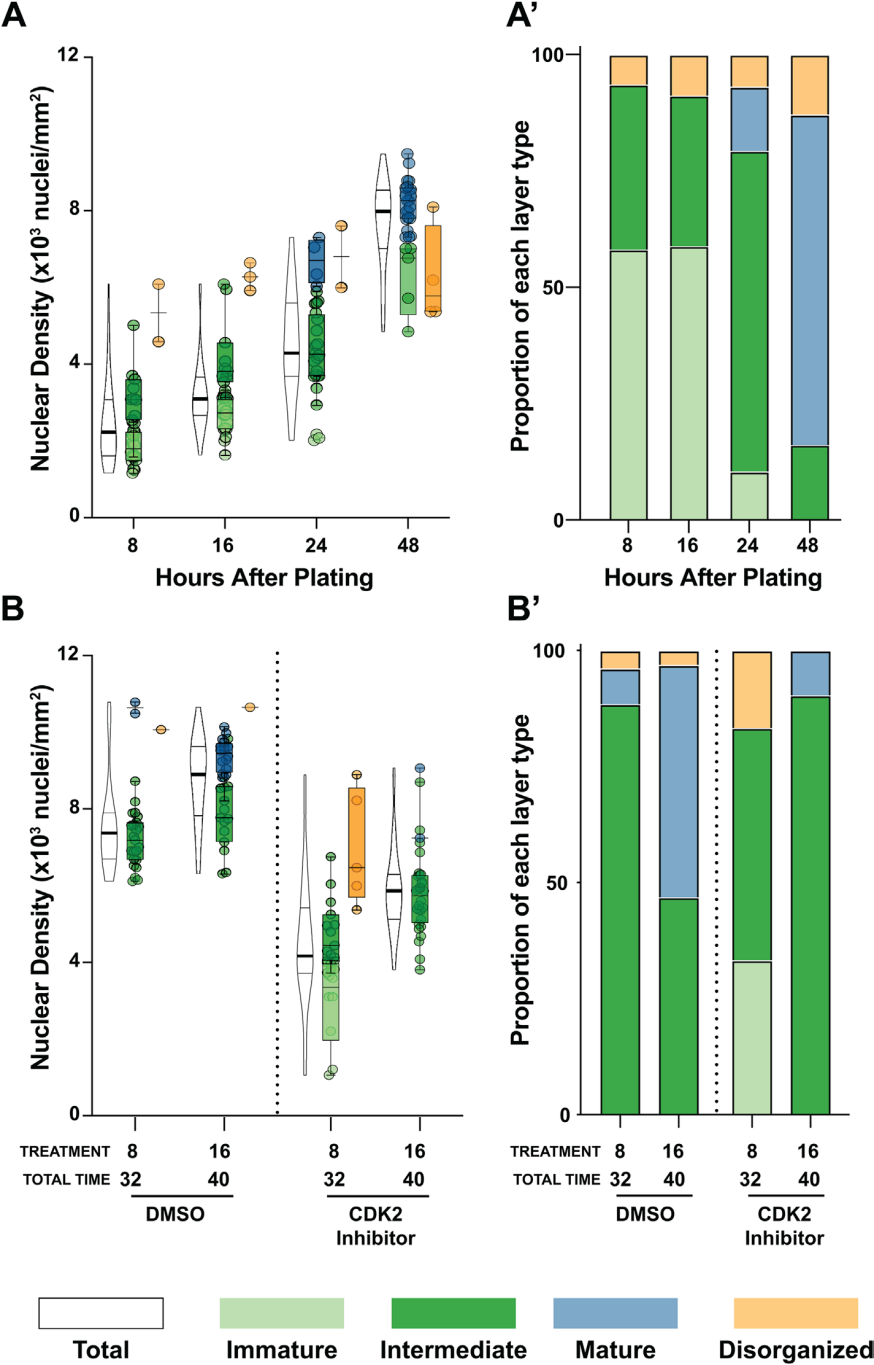
Time spent on the substrate is not sufficient to explain the transition between layer architectures. (A, A′) Immature and Intermediate layers predominate at early time points, whereas Mature layers are the dominant layer architecture by 48 h. (B, B′) Treatment with the CDK inhibitor roscovitine slows both densification and maturation of layer architecture. A total of 200K cells were plated in each experiment. Roscovitine was added at 8 or 16 h and left for 24 h.

Having identified the molecular differences between different monolayer architectures, we next used ALAn to investigate how each architecture arises. By their appearance, the first three architectures suggested a developmental series with cells initially taking on an Immature architecture and transitioning to a Mature architecture. One hypothesis to explain a developmental transition is that time alone could be sufficient to produce a Mature architecture, driven by signaling events such as substrate-initiated basal integrin signaling. β-Integrin signaling is required for polarity establishment in MDCK cells (Lee and Streuli, 2014) and so signaling could contribute to not only molecular differences but also morphological differences. Alternatively, an increase in density could be sufficient to produce a Mature architecture through increased cell–cell interactions, for example, leading to more E-cadherin–based adhesions. Both hypotheses were consistent with our initial data: at a seeding density of 200K cells/cm^2^, we observed layers shifting from predominantly Immature and Intermediate architectures at early time points and low final densities to Intermediate and Mature at later time points with higher densities (Figure 6A). To determine the individual contributions of time and seeding density to monolayer development, we needed to divorce the two factors.

To determine the contribution of time on substrate to monolayer development and subsequently tissue architecture, we restricted monolayer density through the use of roscovitine, a CDK2 inhibitor (Meijer *et al.*, 1997). Roscovitine blocks cell cycle progression at the G1/S transition, thus limiting proliferation and layer densification, allowing us to divorce the contributions of time and density to layer development. When cell division was halted, while some cells could proceed to Intermediate architecture, Mature tissue architecture was largely lost. Time-matched DMSO controls primarily develop into Intermediate/Mature architectures while those treated with roscovitine were found to be Immature/Intermediate (Figure 6B). We also considered whether the development of the actin brush border, a feature of functional epithelia (Christensen *et al.*, 2012), was influenced by the amount of time spent on the substrate. Cells in Immature layers had the same actin profile regardless of layer age (though a caveat in interpreting this result is that individual cell age is not determined) (Supplemental Figure S6, A and B). Thus, time spent on the substrate is insufficient to allow cells to develop Mature monolayer architecture.

**FIGURE 7:**
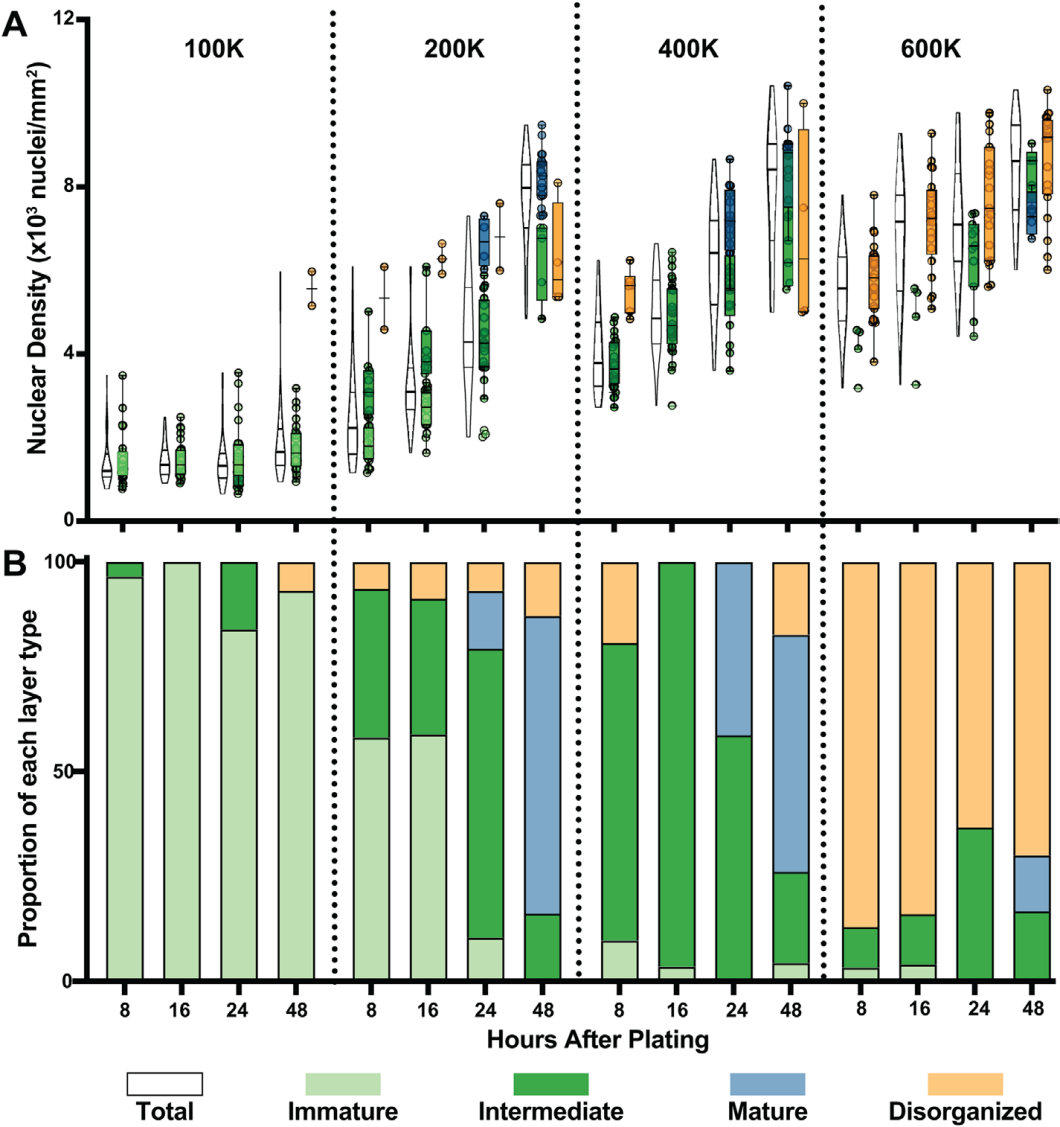
Architecture development at different plating numbers. (A) Immature and Intermediate layers are observed at lower densities, while Mature layers develop at high densities. Seeding at a high density (600K) results primarily in disorganization. (B) Proportions of each layer type found at the four different time points and seeding densities shown in A.

To study the contribution of cell density to layer architecture, we first varied the number of seeded cells. When we reduced seeding density to 100K cells/cm^2^, we found that Immature layers predominated at all time points (Figure 7, A and B), as cells remained at densities similar to those at the earlier time points under our standard seeding conditions. At seeding densities of 200K and 400K cells/cm^2^, most layers are classified as Intermediate at 8 and 16 h, while Mature layers develop at later time points. As predicted by transitions driven by increasing cell density, we do see an increase in the proportion of Intermediate layers at the earlier time points of 8 and 16 h, relative to cells seeded at 100K cells/cm^2^, but Mature layers did not appear at earlier time points; they were still restricted to 24 h onward (Figure 7B). Thus, the architectures match the observed cell densities (Figure 7, A and B). However, if architecture relies solely on an increase in density, further increasing the seeding density should result in Mature architectures at earlier time points. Surprisingly, however, when we increased the seeding density to 600K/cm^2^, rather than an increase in Mature architectures, we observed predominantly Disorganized architectures (Figure 7, A and B). This suggests that the formation of a Mature architecture, while density dependent, does not solely rely on cell density. Together, these results indicate that Mature architectures are a product of an increase in both time and density.

As architecture relies on densification, we hypothesized that reducing cell density of an already formed monolayer would result in a reversal in architecture. To test this hypothesis, we treated monolayers with the drug etoposide, which has previously been used in MDCK cells to induce apoptosis and reduce cell density (Andrade and Rosenblatt, 2011). Upon manual verification of segmentation, we found that pyknotic nuclei were being included (Supplemental Figure S7A). We therefore set a stricter cutoff of 1 SD below the mean volume (Supplemental Figure S7A′).

**FIGURE 8:**
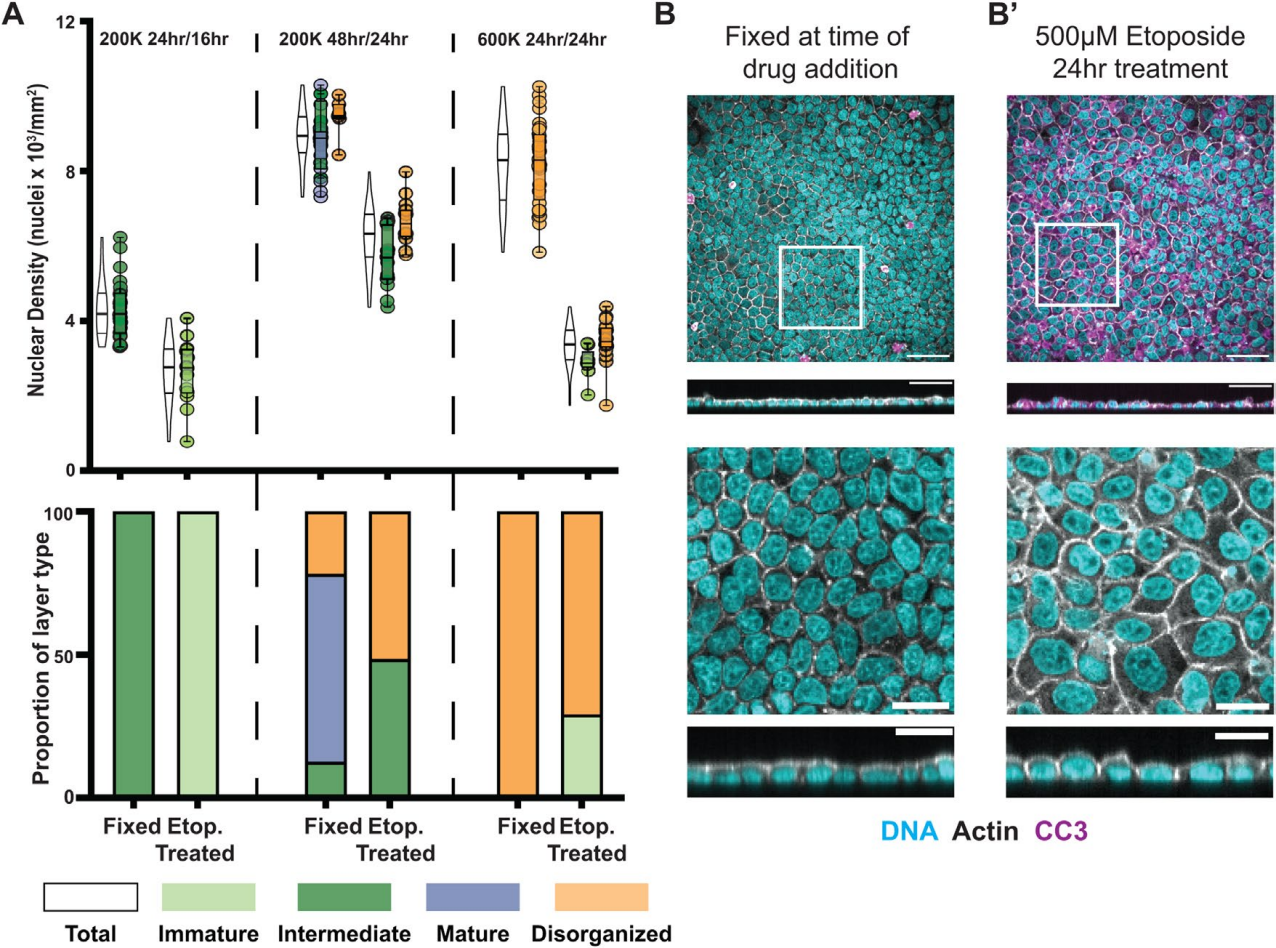
De-densification can promote layer reversal. (A) Layers of different architectures treated with etoposide show architectural reversal. When Intermediate layers are treated with etoposide, cells take on an Immature architecture. Likewise, when Mature layers are treated, they take on an intermediate architecture. Meanwhile, treating Disorganized layers with etoposide forms few organized layers; most remain Disorganized. (B) Representative image showing a Mature layer fixed at the time of treatment in comparison to an etoposide-treated layer classified as Intermediate (B′).

We first allowed 200K cells to develop over a 48 h period to produce primarily Mature architectures (based on data in Figure 6). We then either fixed these monolayers or treated them with dimethyl sulfoxide (DMSO) or etoposide and incubated them for a further 24 h. As expected, Mature monolayers predominated in the fixed controls, with an average density of ∼8.9 cells × 10^3^/mm^2^ (Figure 8, A and B). Upon etoposide treatment, cell density decreased to an average of ∼6.3 cells × 10^3^/mm^2^. Mature architecture was lost, and it was replaced by cells with an Intermediate or Disorganized architecture (Figure 8A). After etoposide treatment, cells display the same characteristics as the Intermediate architectures in our previous experiments; the apices of cells were domed rather than flat, while cell areas increased and circularity decreased (Figure 8B; Supplemental Figure S7, B and C).

We also treated Intermediate layers with etoposide. One caveat to reducing the density of Intermediate layers is that many cells die over longer periods, resulting in images in which there are gaps between cells (Supplemental Figure S7D). We therefore reduced the incubation time to 16 h in etoposide and ensured that we excluded any images with gaps in them. We found that treating Intermediate layers with etoposide for 16 h results in a reversal back to Immature architecture (Figure 8A), with cells spreading on the substrate and reverting to their “fried egg” morphology. This led us to conclude that reducing density can result in layer architecture reversal. We also examined the effect of reducing the cell density of cells that began with a Disorganized architecture. Seeding 600K for 24 h results in Disorganized architectures, as expected, but treatment with etoposide for 24 h did not restore Mature architecture; most remain Disorganized, with a few lower-density images classified as Immature (Figure 8A). We therefore conclude that cells that begin in a Disorganized configuration are likely to remain Disorganized, even at lower densities.

### Cells can stably attach and aggregate above an organized monolayer

**FIGURE 9:**
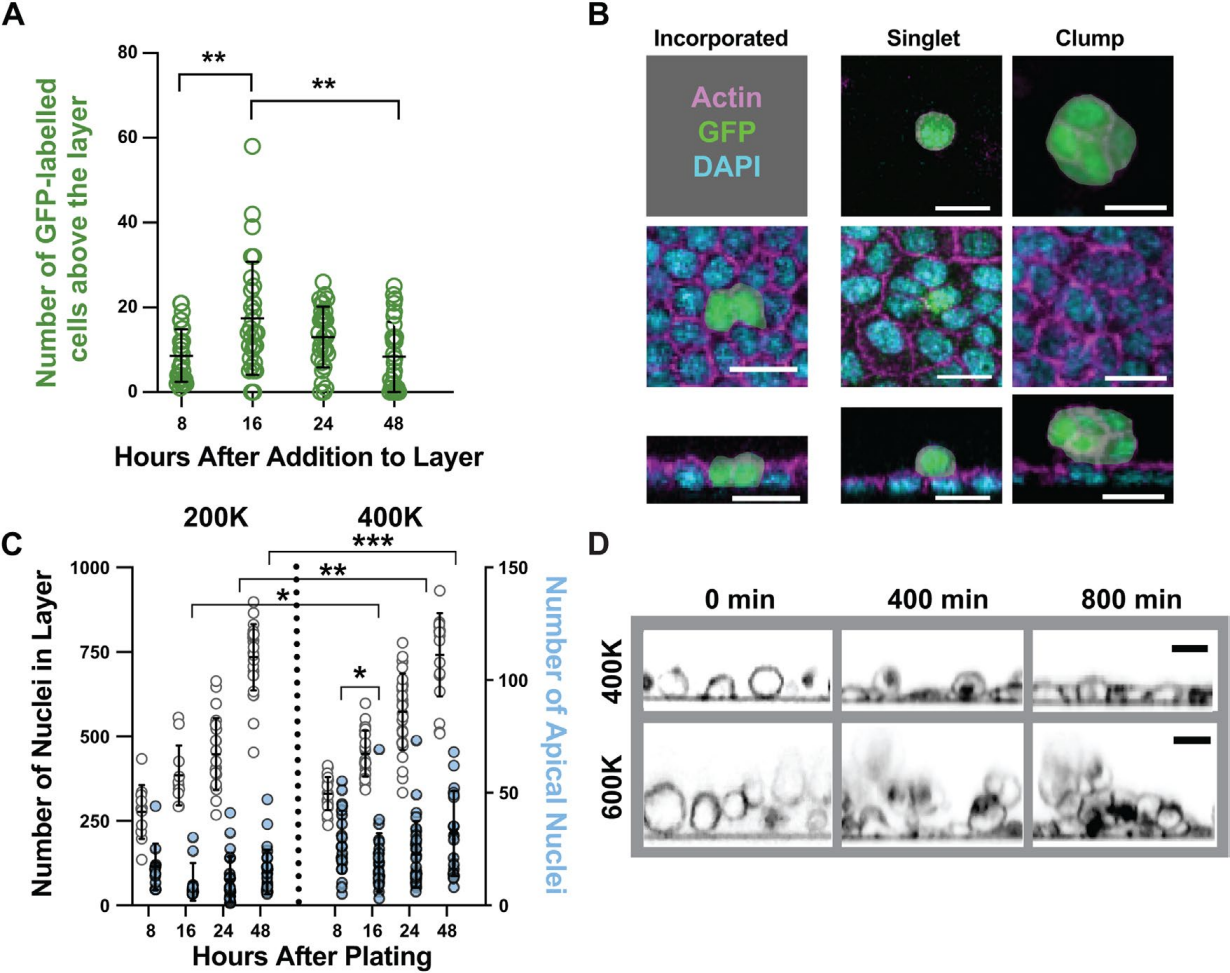
Cells can stably attach and aggregate above an organized monolayer. (A) Seeding GFP-labeled MDCK cells on a preexisting layer shows that cells exogenously introduced to a monolayer can attach apically. *P* values left to right: *p* = 0.0017, *p* = 0.0026. (B) GFP–labeled MDCK cells added to a preexisting monolayer can be found in different configurations: singlets, clumps, or incorporated. (C) The number of apically positioned nuclei in Intermediate or Mature layers increases with seeding density. (D) Live imaging of MDCK cells at low and high plating density (Supplemental Movies 4 and 5). Cells were imaged for 13 h after seeding. Cell membranes are marked with CellMask Orange. All statistics unpaired, two-tailed Student’s *t* test.

As a further test of ALAn’s utility, we next explored how Disorganized architectures develop. A straightforward possibility is that disorganization is caused by cells that land after the substrate has been covered. To test this hypothesis, we allowed 200K cells to develop over a 24 h time period, at which point architectures are primarily Intermediate, and then added GFP-labeled cells. We found that GFP+ cells attach apically to the existing layer, peaking in number at ∼16 h after addition. This number declines gradually over the next 32 h, suggesting that the GFP+ cells either become part of the existing layer or undergo detachment and/or cell death (Figure 9A). While we observed GFP+ cells that had been incorporated into the underlying layer, these cells were too rare to accurately count (Figure 9B). In contrast to the apically positioned GFP+ cells, which were frequently observed in aggregates apical to the layer, the incorporated GFP+ cells were morphologically indistinct from their nonlabeled neighbors (Figure 9B). Thus, cells can form stable attachments to the apical surface of an underlying cell monolayer.

**Figure:** Movie S4 **400K Plating Layer Formation** By 400 minutes, cells plated at 200K condition have spread to form an Intermediate architecture. Movie taken at 10 minutes / frame shown at 10 frames / second.

**Figure:** Movie S5 **600K Plating Layer Formation** By 400 minutes, cells plated at 600K have aggregated to form a Disorganized architecture. Movie taken at 10 minutes / frame shown at 10 frames / second.

We next asked whether apically positioned cells are observed in our organized layers. We considered this likely, because not every cell in a plating suspension is expected to land on the substrate at the same time. In addition, we would expect the number of apically positioned nuclei to correlate with seeding number. Because ALAn can distinguish apically positioned nuclei, we used it to quantify them in our Intermediate and Mature layers. Both predictions were confirmed (Figure 9C; Supplemental Figure S8A). Apically positioned cells were seen at every time point without substantial variation in number as the layers became more mature—the small dip in the number of apical nuclei between 8 and 16 h likely reflects the fact that a small number of cells (∼2%) at the 8 h time point are connected to the substrate but have not yet finished settling down (Supplemental Figure S8B). These cells are indistinguishable from their neighbors by the 16 h time point (Supplemental Figure S8C).

We also considered whether some of the apically positioned nuclei originate inside the layer as a consequence of misplaced division products. While we do not discount this possibility, proliferation is not the major driver of apically positioned nuclei in our experiments, because their number was not significantly decreased in layers treated with roscovitine (Supplemental Figure S7D). Together, the above results reveal that 1) cells can form stable attachments to the apical surface of an underlying layer; 2) apically attached cells can form aggregates on top of an organized layer (Supplemental Figure S8E); and most importantly, 3) the addition of cells to an extant layer is not sufficient to cause layer disorganization

However, the aggregates that we observed have an appearance akin to that of the Disorganized architecture recognized by ALAn, suggesting the possibility that disorganization arises when cells can contact each other but not the substrate. We performed live imaging to probe this possibility. At the lower seeding density (400K cells/cm^2^), cells follow a stereotypical pattern: a cell reaches the substrate, undergoes spreading, and then develops cell–cell contacts. Live imaging at 600K cells/cm^2^ reveals that cells that adhere to the substrate are quickly covered by additional cells and never form an organized underlying layer, resulting in a Disorganized architecture (Figure 9D). This led us to revise our hypothesis; disorganization is the result of an excess of cells aggregating while the tissue is in a fluid regime.

### Tissue fluidity correlates with the capacity to reorganize in the apical-basal axis

**FIGURE 10:**
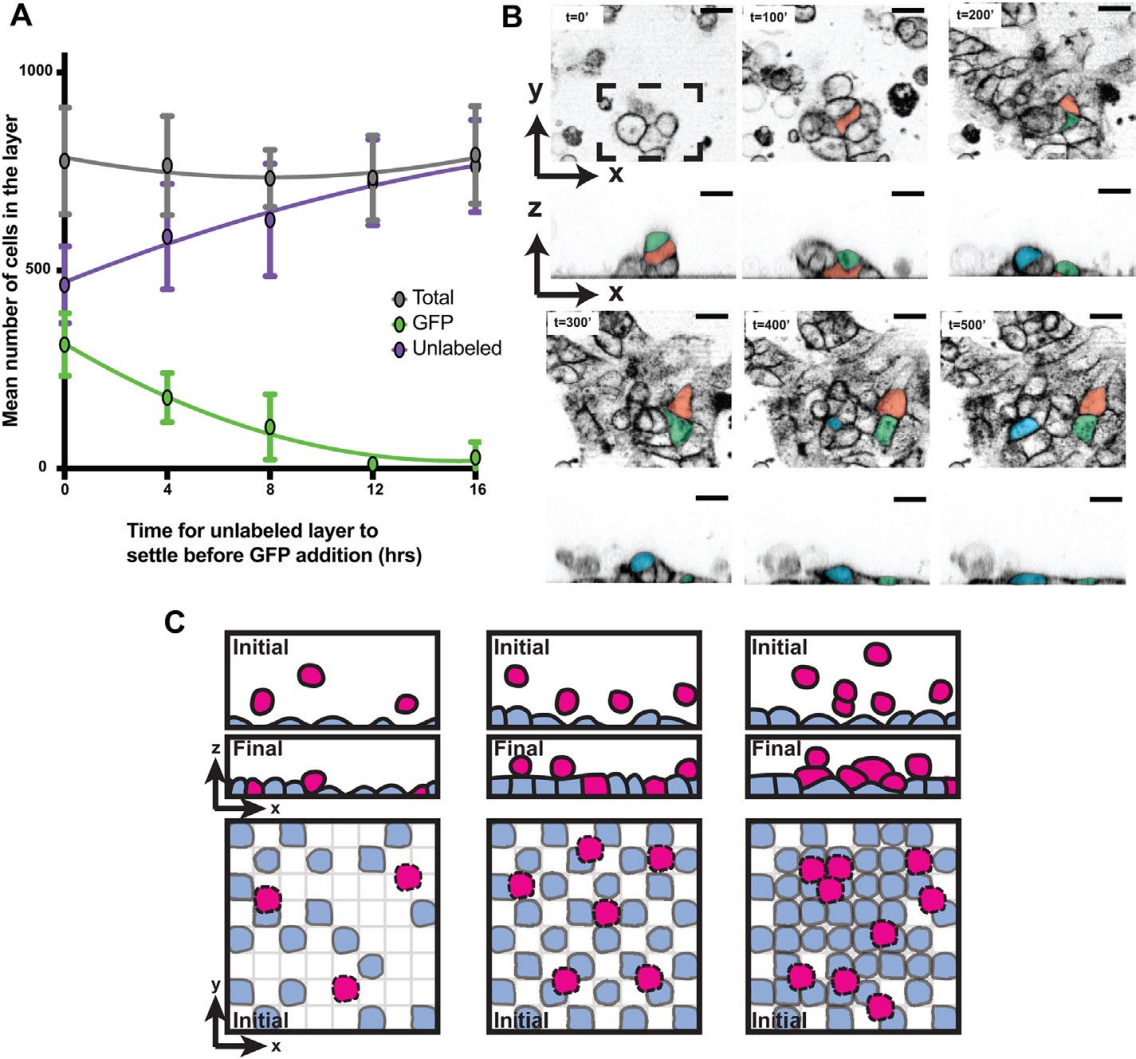
Cell incorporation decreases as cells form substrate connections. (A) The mean numbers of unlabeled (purple) and GFP-labeled (green) cells for each time point were plotted, along with total cell number (gray). Cells are less likely to be incorporated into the layer as early as 4 h postseeding. (B) Time course of cells seeded as clumps rather than dissociated cells (Supplemental Movie 6). Cells positioned apically at time point 0 (false-colored cells) move basally to form substrate connections as underlying cells spread. Scale bar = 20 µm. (C) Architecture is governed by cell–cell and cell–substrate adhesion. At a low seeding density, initial cells will adhere to the substrate and spread (blue). Cells that subsequently arrive (pink) have sufficient substrate available and form cell–cell adhesions (left). As seeding density increases, there is less substrate available for the late-landing cells, resulting in an increase in cells apically positioned on an organized architecture (middle and right).

A prediction of our revised hypothesis is that as cells spread to cover the substrate and begin forming cell–cell contacts, their ability to reorganize in the apical-basal axis decreases rapidly. To test this hypothesis, we investigated how layers develop by first seeding unlabeled MDCK cells and then adding GFP-labeled cells at later time points in 4 h increments. After a total period of 24 h cells were fixed and stained, and we combined the use of ALAn and manual counting to determine the total number of cells within the layer and the fraction of these that were GFP+ (Supplemental Figure S9A). If unlabeled and GFP+ cells are seeded simultaneously, the resulting monolayers contain approximately equal numbers of each cell type (Figure 10A). However, the proportion of GFP+ cells attached to the substrate correlates inversely with the time of addition and is negligible (∼3%) if these cells are added 16 h after the unlabeled cells (Figure 9A). This trend is also observed at a lower initial seeding density (Supplemental Figure S9B). Together, these results suggest that cells initially prioritize spreading on the substrate and that as the substrate becomes covered, the likelihood of subsequent cell incorporation decreases.

**Figure:** Movie S6 **Cell Clump Landing on Substrate** A live XZ reconstruction of cells landing on a substrate. These cells undergo reorganization in the apical-basal axis to go from a clump into a monolayer. Movie taken at 10 minutes / frame shown at 10 frames / second.

To test whether substrate availability is sufficient for apically positioned cells to organize, we seeded clumps of cells rather than dissociated cells and visualized the outcome using live imaging. If substrate availability is sufficient, we hypothesized that cells initially positioned apically within the clump would be able to move down and contact the substrate. Alternatively, the cells might remain in clumps and never organize in the apical-basal axis. We found that the first cells in the clump to contact the substrate spread as expected. The cells positioned higher up in the clump reorganize downward, undergoing rearrangements and neighbor exchanges as they do so, resulting in all cells adhering to the substrate by the final time point (Figure 10B).

Together, these data suggest that cell rearrangements along the apical-basal axis differ from those in the tissue plane; rearrangements in the apical-basal axis decrease rapidly as soon as the substrate is covered, while cells in the tissue plane can still undergo rearrangements until they reach an intermediate state. Thus, the increase in disorganization that we observed at higher seeding densities (Figure 7) is likely the result of a lack of free substrate. At a seeding density of 600K cells, the substrate is covered immediately due to the sheer number of cells, resulting in a high proportion of cells being unable to form substrate connections. As a result, those positioned above cannot establish polarity without substrate contact, while the cells in contact with the substrate do not have a free apical surface. They instead form contacts with the apically positioned cells, thus becoming indistinguishable from them. Intriguingly, even when the density of a Disorganized architecture is subsequently reduced, the cells predominantly remain Disorganized (Figure 8). We therefore conclude that while monolayer maturity is at least in part density dependent, the formation of a Mature monolayer relies on additional processes not identified in this study.

Our results, summarized visually in Figure 10C, suggest a model in which cells are in a “race to the bottom.” When free substrate is available, cells are more motile and exhibit a fluid-like behavior. After substrate covering, cell–cell adhesion predominates, resulting in the formation of lateral surfaces for cells on the substrate, while those above adhere to the underlying developing monolayer.

## DISCUSSION

We characterized biologically distinct tissue architectures that form in epithelial cell culture and developed a novel tool to distinguish them. We anticipate that this tool will be broadly applicable for cell culture studies of epithelial biology to ensure consistency of methodology across studies. Our study also addresses the question of how monolayer architecture is achieved and in particular whether the distinct viscoelastic material transitions that have been described with respect to the tissue plane extend to the apical-basal axis. Our findings show that MDCK cell tissues are fluid in the apical-basal axis when there is available basal substrate for cells to expand into, but this fluidity ceases rapidly as this substrate availability decreases. Thus, there is a jamming transition for epithelia in the apical-basal axis that is dependent on the ability of cells to contact the basal substrate.

### Why use ALAn?

Our work shows that obvious experimental parameters, including plating density, confluency, and time spent on the substrate, are imperfect indicators of layer architecture, which can vary substantially across a culture well in multiple cell types. As a result, single *xz* frames may be insufficient to determine layer architecture at mesoscale. Past studies have had to rely on manual frame selection as a basis for comparison between experimental conditions (for example, Schoenenberger *et al.*, 1991; Meder *et al.*, 2005; Jenkins *et al.*, 2015). Here we introduce our image analysis toolset, ALAn, as an improvement over manual determination.

ALAn uses just two inputs to determine epithelial architecture: nuclear positional information and actin intensity. These inputs are obtained through stainings regularly used in epithelial biology, that is, DAPI for DNA and phalloidin for actin, meaning that the user can perform experiments in their usual manner with no additional reagents required. This makes our tool easily accessible to the community, without the need for additional/modified steps in already established protocols.

ALAn also facilitates comparison across studies. Our analysis of the literature reveals that MDCK monolayers that we would define as either Intermediate or Mature have been used for studies investigating epithelial architecture (for example, Fouchard *et al.*, 2020; Harmand *et al.*, 2021). These architectures have different material and polarity properties, and therefore care must be taken in drawing comparisons between them.

### In vitro epithelial cell cultures undergo architecture transitions that correlate with cell density

Epithelial cell cultures exhibit distinct architectural transitions that correlate with biological polarity transitions during monolayer development. Architecture transitions similar to those observed in our studies take place in the zebrafish embryo. A series of divisions densifies the developing tissue composed of blastomeres. At the two-cell stage, adjacent blastomeres do not adhere. By the eight-cell stage, blastomeres take on a cobblestone appearance with domed apical surfaces and begin to develop cadherin-based adhesion along their lateral surfaces. Blastomeres at the 32-cell stage are tightly compacted with mature cadherin adhesions and demonstrate uniform, flat apical surfaces (Jesuthasan, 1998). In light of our cell culture results, these observations suggest that densification correlates with epithelialization in vivo*.* This raises the question of whether densification participates in developmental processes such as MET (Kim *et al.*, 2017).

Conversely, the correlation between zebrafish embryogenesis and MDCK development raises the question of how cell–cell adhesion affects the development of the architectures described here. MET in processes like kidney development (Shah *et al.*, 2004) or wound healing (Poujade *et al.*, 2007) is dependent on cell–cell adhesion. If adhesion alone could account for architecture transition, we would expect to have seen evidence of this in the roscovitine experiment. This, however, does not rule out the possibility that adhesion influences the role of density in layer transition.

### Density-independent mechanisms that could influence layer architecture

While we focus primarily on densification in this study, many other mechanisms can influence tissue architecture. While densification results in a transition from Immature to Intermediate to Mature architectures, de-densification of a Disorganized architecture does not result in a Mature layer. This suggests that while densification plays an important role, other processes are required in order to develop a Mature architecture. Factors such as cell–cell and cell–substrate adhesion strength along with tissue level tension, while intricately linked to density, can independently contribute to a fluid-to-solid transition and therefore could influence epithelial maturity (Lawson-Keister and Manning, 2021). For example, decreasing cell–cell adhesion is known to increase fluidity in the tissue plane. One could speculate that decreasing cell–cell adhesion could also increase fluidity in the apical-basal axis as cells contacting the substrate would not form as strong connections between one another. The decrease in adhesion could create more gaps between cells on the substrate, allowing those above to enter the monolayer. Conversely, we have previously shown that cell reintegration following mitotic cell daughter displacement is an active process involving the lateral adhesion machinery and spectrin-based cytoskeleton (Bergstralh *et al.*, 2015; Cammarota *et al.*, 2020). An activation of this integration machinery could increase tissue fluidity in the apical basal plane by actively drawing cells into a tissue monolayer.

Tissue tension and its effects on monolayer architecture is another avenue to explore. As cells are connected through junctional complexes, they can sense fluctuations in tension across the tissue. For example, recent work using MDCK monolayers has shown that cell division results in changes in junctional tension, which can affect the morphogenesis of the monolayer and increase fluidity in the tissue plane (Devany *et al.*, 2021). This increase in fluidity can occur independently of an EMT-like change in the adhesion or tension profile of epithelial cells (Mitchel *et al.*, 2020).

Cell–substrate adhesion through integrins, for example, could influence not only architectural transition but monolayer organization in MDCK cells. Modeling suggests that substrate adhesion can alter the ability for epithelial monolayers to regulate apical-basal architecture (Galle *et al.*, 2005). Given the implications of both cell–cell and cell–substrate adhesion in the development of monolayer architecture, a more complete model of architectural development may include the balance of physical forces on a cell from layer densification, adhesive interactions, and signaling pathways.

### How can exogenous cells become part of the underlying monolayer?

In our study we find that Disorganized architectures are a consequence of the lack of substrate availability. As cells cover a substrate and begin to form cell–cell connections, they quickly create a barrier to exclude exogenous cells. However, we do find that on occasion, cells can be incorporated into the existing monolayer. This raises the question of what makes these cells distinct from those unable to be incorporated into the monolayer. Integrations can be important morphogenetic or regulatory processes in epithelia. During *Caenorhabditis elegans* morphogenesis, anchor cell invasion requires an apically positioned cell to invade the underlying basement membrane and become part of the underlying monolayer (Hagedorn and Sherwood, 2011). This process is closely linked to the cell cycle; in order to invade the basement membrane, the anchor cell is required to be arrested in the G1 phase of the cell cycle (Matus *et al.*, 2015; Lattmann *et al.*, 2021). Other apical-basal morphogenetic processes such as epithelial cell reintegration are also linked to cell cycle dynamics. Daughter cell position is initially determined by the orientation of the metaphase spindle, which sets up the direction of division and is typically aligned in epithelia so that both daughter cells appear within the tissue plane (reviewed in di Pietro *et al.*, 2016; Bergstralh *et al.*, 2017). Misorientation of the spindle can cause a daughter cell to be positioned outside the monolayer, but this does not lead to tissue disorganization due to cell reintegration (Bergstralh *et al.*, 2015; Wilson and Bergstralh, 2017; Cammarota *et al.*, 2020). The same mechanism driving anchor cell invasion and cell reintegration could be allowing MDCK cells to overcome the loss of fluidity in the apical-basal axis. Furthermore, the presence of apically positioned cells could result in morphological changes in the cell(s) on which they are attached. Recent work has shown that compression of cells is sufficient to induce a density-independent solid-to-fluid transition (Park *et al.*, 2015). If a similar process occurs in MDCK cells, the apical cells could induce a temporary solid-to-fluid transition to allow for apically positioned cells to integrate into the layer. Understanding how these apically positioned cells occasionally become part of the monolayer is a direction for future work.

## MATERIALS AND METHODS

Request a protocol through *Bio-protocol*.

### Reagents

A list of reagents used in this study can be found in Supplemental Table 1.

### Cell culture and immunostaining

Cells were passaged 1 d before seeding on eight-well collagen-coated slides. Media was changed every 24 h. After the allotted time period, the media was removed, the cells were washed with Dulbecco’s phosphate-buffered saline (dPBS), and the cells were fixed using ∼4% formaldehyde, 2% PBS-Tween for 10 min. Three washes (10 min each) in PBS–0.2% Tween were carried out between fixation and stainings. Primary antibodies were added at 1:500 dilution as were secondary antibodies. Fluorescein isothiocyanate phalloidin was used to stain actin. Vectashield plus DAPI was then added to the wells. A concentration of 1.5 µg/ml roscovitine reduced the final cell number without obvious toxicity/cell death. A concentration of 500 µM etoposide was used for either 24 or 16 h, depending on the initial cell density. Exogenous MDCK cells were mosaically transfected using a CMV GFP vector (pLenti CMV GFP Blast [659-1] was a gift from Eric Campeau and Paul Kaufman [UMass Chan Medical School, Worcester MA; Addgene plasmid #17445; http://n2t.net/addgene:17445; RRID:Addgene17445]). GFP-positive cells were then selected for and maintained in 10 µg/ml blasticidine. Fifty thousand GFP cells were seeded onto an existing monolayer.

### Imaging and segmentation

Cells were imaged on an Andor Dragonfly Spinning Disk Confocal microscope using a 40×/1.15 water objective. Confocal stacks were taken beginning beneath the bottom of the chamber slide where no actin signal could be detected and ending above the layer when no more actin signal could be detected, taken at a z-spacing of 0.23 µm. To control for differences between culture wells, the same 16 spots were imaged in each well. After image acquisition, the image analysis software Imaris was used to segment all of the nuclei within the images as detailed in ALAn. In our study, the nucleus diameter is set to 7.3 µm and smoothed with a filter width of 0.73 µm; nuclei are split by Seed Points with the quality factor set to 4; the intensity threshold is set to 120 for most of our images. Images with different cell types (e.g., Caco2 cells) and live imaging segmentation parameters were determined on a per image basis. Seeding number and time point were blinded to the experimenter for our large data set with various densities and time points.

Live imaging was performed on a Leica SP5 confocal with a 40× oil objective, and images were collected with LAS AF. Z-stacks were taken at 10 min intervals, and movies were processed using a Gaussian blur with FIJI. MDCK cells were passaged 24 h before imaging. The next day, cells were seeded onto collagencoated Ibidi slides in a volume of 200 µl and immediately stained with 10 µl of a 1:500 dilution of CellMask Orange. A spacing of 1 µm was used during z-stack acquisition, and a stack was taken every 10 min overnight.

### Live movie autocorrelation function

To measure the total change in an image over time, we generated autocorrelation functions that compare time points in an image separated by a certain amount of time. The logic behind this analysis is that between two successive time points we do not expect to see much change, but between the first and last images we might see significantly more. This autocorrelation function will quantify the rate at which images “decorrelate,” meaning that they become unrelated. The correlation strength is a –1 to 1 scale where –1 is perfectly anticorrelated, 0 is uncorrelated, and 1 is perfectly correlated. The correlation time is the amount of time between the two frames being compared. Each data point is the average of all correlation strengths between all pairs of frames separated by the given correlation time. The following function describes the autocorrelation function:




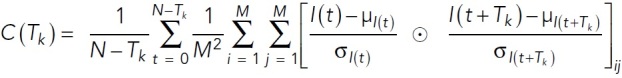




where *C* is the correlation strength, *T_k_* is the correlation time, *N* is the number of time points in a movie, *M*^2^ is the number of pixels in an image of the movie, 
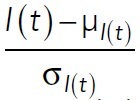
 is the normalized image (subtract average pixel intensity and divide by the SD) at time point *t*, 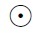 is an element-wise multiplication, and the indices *i* and *j* correspond to the sizes of the images. Additionally, the curves were further analyzed by fitting the autocorrelation to an exponential decay to estimate the amount of time it takes for the cells within an image to be decorrelated. The given values for decay are the half-lives given the best fit rate.

### Actin and aPKC polarization ratios

The actin polarization ratio was measured on a per-cell basis by first duplicating a circular portion of the z-stack through the center of a cell’s nucleus, making sure not to include cell–cell borders. This cylindrical core was then summed through the *XY* planes and normalized to create an intensity versus z plot as done for the entire actin intensity in ALAn (Supplemental Figure S2B). The actin plots made from the cylindrical core have two peaks, corresponding to the apical and basal surfaces (as opposed to ALAn’s plots, which are primarily one-peaked due to the lateral surfaces).

### ALAn availability

The ALAn tool was written in Python in a Jupyter notebook and is available for download on the Bergstralh-Lab GitHub repository: https://github.com/Bergstralh-Lab.

## Abbreviation used:

ALAn: Automated Layer Analysis.
