# Supplementary material for: A novel tool for the unbiased characterization of epithelial monolayer development in culture
Source: Mol Biol Cell. 2023 Mar 7;34(4):ar25. doi: 10.1091/mbc.E22-04-0121 (PMC10092640; doi:10.1091/mbc.E22-04-0121)
Supplement: Supplementary file 7 [file mbc-34-ar25-s001.pdf]

Supplementary Materials
Molecular Biology of the Cell
Dawney *et al*.

Supplemental Figure 1: Development and verification of Automated Layer Analysis.

A) Segmented objects smaller than 1.5*standard deviation of the mean volume are removed from the analysis. A') Nuclei are observed above 1.5*standard deviation above the mean volume, so no upper cutoff is set. B) Nuclei positions are binned across Z in 1 µm increments with an average across 3 µm producing the gaussian fit. C) The peak of the actin intensity does not correspond to the top of the layer and so the layer bounds are set to be 60-80% of the peak intensity, dependent on density. D) Comparison between manual (performed by 4 researchers independently) and automated determination of layer boundaries. p-values from left to right: $p = 0.2722$, $p = 0.1512$, $p = 0.6850$, $p > 0.9999$, $p = 0.5720$, $p = 0.3005$, $p = 0.9134$, $p = 0.9248$. E) A mean deviation cut-off of 5 determines whether the nuclei follow a single (1 peak) or double (2 peak) Gaussian distribution. F) If the nuclei follow a single Gaussian distribution, the width of the peak vs. position of the peak determines organization. G) Mature monolayers display a two-step growth to the peak intensity reflected in the actin plot. G') The first derivative of the actin plot shows 2 peaks, with the apical peak being more prominent than the peak reflecting lateral surfaces. H) Intermediate layers have mono-peaked actin intensity plots therefore the derivative of Intermediate layers either has one peak (H') or the ratio of the right peak to the left peak is less than one. I) Manual counts of apically-positioned nuclei match with ALAn's counts. Manual counts performed blind by 4 researchers independently. X-axis categories represent different images. $p = 0.0011$, $p = 0.0708$, $p = 0.7120$, $p = 0.0764$. All statistics unpaired, two-tailed Student's t-test.

Supplemental Figure 2: Quantification of Layer Fluidity

A) Schematic representing how the quantification in B was performed. All movie frames separated by a given correlation time are compared and averaged to find the correlation strength. B) Supplemental Movies 1-3 are analyzed using the autocorrelation function and plotted together. The Mature layer decorrelates the slowest, given the blue curve remains at a higher correlation strength throughout the comparisons. B') The decay rate is measured for each movie by fitting a decaying exponential to each autocorrelation plot. The half-life for the layer types from left to right are 287, 424, and 754 minutes respectively.

Supplemental Figure 3: Markers of polarity and adhesion in different layers.

A) aPKC is observed at all cell-cell borders in the disorganized regions. B) ZO-1 signal at the cell-cell border (position 0) is sharper in a Mature layer cell than an Immature layer cell.

Supplemental Figure 4: Live MDCK, MCF-7 and Caco2 analysis with ALAn

A) Live imaging of MDCK cells stained with Hoechst and SiR-Actin produces promising plots for classiying live samples.B-E) Layer characteristics of MCF-7 cells are similar to those seen in MDCK cells. B) Densities of each layer type found in MCF-7 cell cultures cell area decreases as layers mature (C) while layer height (D) and cell circularity (E) increase as layers transition from Immature to Mature. Immature n = 5, Intermediate n = 20, Mature n = 10. E) . n = 7 Immature, 17 Intermediate, 10 Mature, 18 Disorganized. F-I) Example plots from Caco2 monolayer cultures representing Immature (F), Intermediate (G), Mature (H) and Disorganized (I) architectures. A prominent basal peak can be seen in all architectures.

Supplemental Figure 5: Smaller regions accurately represent overall image density

A) Densities found within a range of box sizes from 25 µm x 25 µm up to the full image (~300 µm x 300 µm) show that while the spread of densities is greater in the smaller regions, the mean does not change meaning the density found in a 25 µm x 25 µm box accurately represents that of the full image. B) Representative image showing how some of the regions within the 25 µm x 25 µm boxes have a density of 0 cells $x10^3/mm^2$. C) Plotting the densities for 25 µm x 25 µm and 100 µm x 100 µm against the full image density shows correlations of ~1, again showing the densities in smaller regions are representative of the whole image.

Supplemental Figure 6: Actin profiles of cells in Immature layers.

A) Single cell actin/aPKC plots were made by taking a circular section through center of the nucleus in the XY plane. The X and Y components are summed, resulting in an intensity vs. Z plot which captures apical and basal surfaces while avoiding lateral surfaces. B) Cortical actin distributions are independent of layer age. Asymmetry is the same in Immature cells at every timepoint examined, including in Roscovitine treated cells. Example plots are shown.

Supplemental Figure 7: Characteristics of Etoposide treated monolayers.

A) Pyknotic nuclei are seen throughout the etoposide treated monolayers which are included in the nuclear plot with a cut-off of 1.5 standard deviations below the mean nuclear volume.

A’) Most of the pyknotic nuclei are excluded when a stricter cut-off of 1 standard deviation below the mean is used. B, C) Characteristics of cells treated with etoposide are as expected. Cell area increases as monolayers reverse to Intermediate (B) while cell circularity decreases (C). D) Intermediate layers treated with etoposide results in some images with gaps between cells. These images were excluded from the analysis. E) Etoposide data from Figure 7 including DMSO controls.

Supplemental Figure 8: Analysis of apically-positioned nuclei.

A) Representative image of a Mature monolayer with a cell on top of the monolayer. B) At 8 hours post seeding, rare (~2%) cells are attached to the substrate but not yet settled into the layer. The nuclei of these cells can be situated above the rest of the layer. Height of the layer (as determined by ALAn) is shown by the dashed line. C) Example of an apically-protruding cell (false colored) as it finishes settling past 8 hours post seeding. D) 200K MDCK cells were plated and allowed to develop for 24hrs, then treated with either DMSO or Roscovitine for a further 24hrs. Comparison between the two conditions shows no significant difference in the number of apically-positioned nuclei. Average densities: DMSO control = 9.22 $x10^3$ nuclei/mm^2; Roscovitine treated = 7.04 $x10^3$ nuclei/mm^2. $p = 0.0683$. E) Aggregates positioned on top of an organized monolayer. All statistics unpaired, two-tailed Student’s t-test.

Supplemental Figure 9: Cell incorporation decreases as cells cover the substrate.

A) Representative image indicating how images were analyzed. Both unlabeled and GFP labeled cells on the substrate were manually counted using the Counter tool in FiJI. B) Cells seeded at a lower density (100K of each labeled and unlabeled) follow the same trend as cells seeded at a higher density.

Supplemental Table 1: Reagents Used in this Study

Reagent	Supplier	Product Code
Collagen IV coated 8-well µ-slide	Ibidi	80822
0.25% Trypsin-EDTA (1x)	Gibco	210200-056
Trypan Blue Stain 0.4%	Gibco	15250-61
Rabbit anti-Ecadherin	Cell signaling	24E10
Rabbit anti-aPKC	Santa Cruz Biotechnology	sc-216
Rabbit anti-ZO1	Abcam	ab96587
Alexa Fluor 633 Goat anti-Rabbit	Invitrogen	A21071
Fluorescein Phalloidin	Invitrogen	F432
Vectashield antifade mounting medium with DAPI	Vector Laboratories	H-1200
CellMask Orange	Invitrogen	REFC10045
DMEM/F12(1:1) (1x)	Gibco	11330-032
Penicillin-Streptomycin (10,000 U/mL)	Gibco	15140-122
Fetal Bovine Serum	Gibco	26140079
Roscovitine	Sigma	R7772-1MG
Etoposide	Sigma	E1383
DMSO	Sigma	D2438-50M
CellMask Actin Tracking	ThermoFisher	A57245
Hoechst 33342	ThermoFisher	H21492
SiR-actin	Cytoskeleton	CY-SC001

SUPPLEMENTAL FIGURE 1

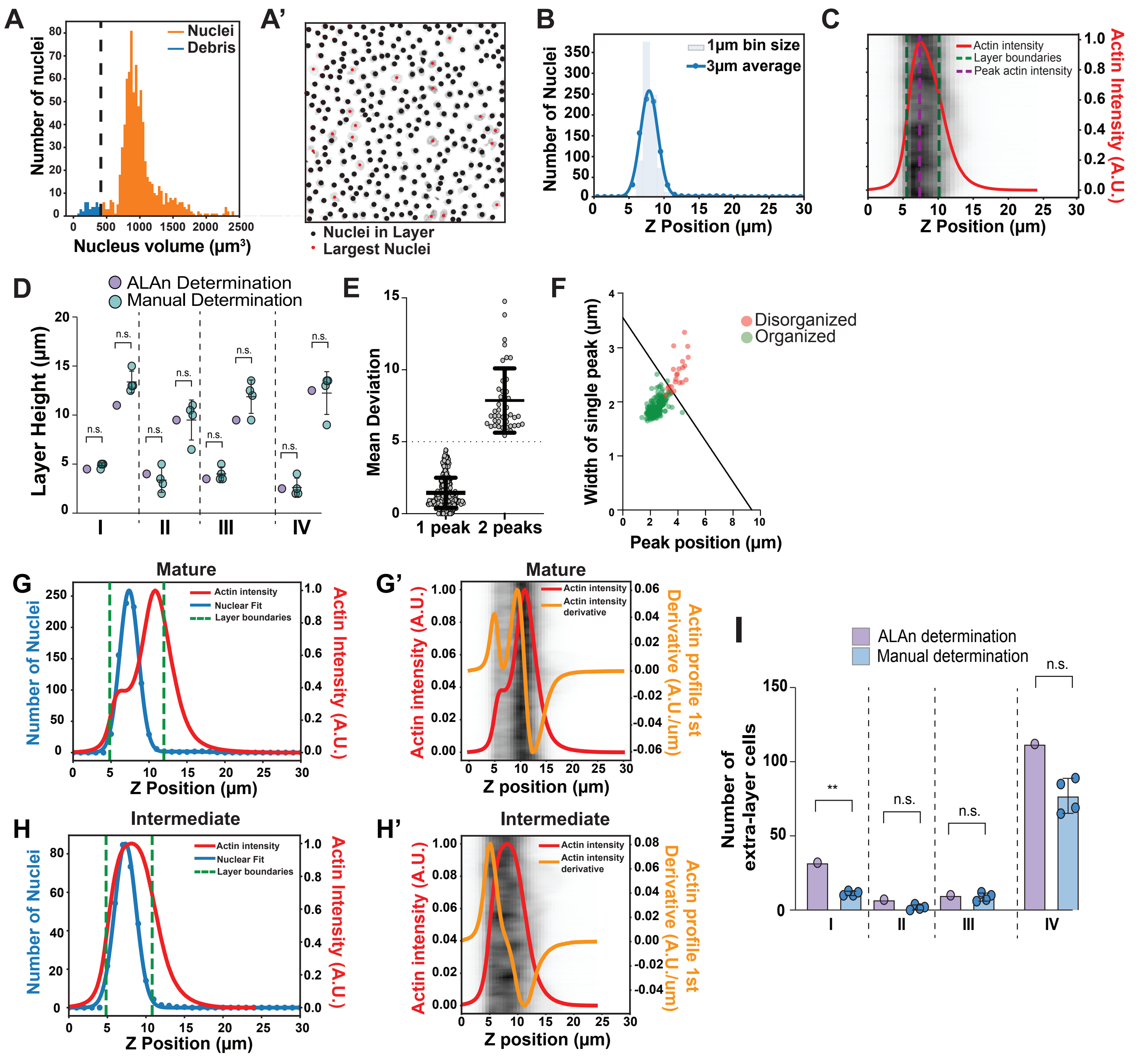

Supplemental Figure 1: Development and verification of Automated Layer Analysis.
A) Segmented objects smaller than 1.5*standard deviation of the mean volume are removed from the analysis. A') Nuclei are observed above 1.5*standard deviation above the mean volume, so no upper cutoff is set. B) Nuclei positions are binned across Z in 1 µm increments with an average across 3 µm producing the gaussian fit. C) The peak of the actin intensity does not correspond to the top of the layer and so the layer bounds are set to be 60-80% of the peak intensity, dependent on density. D) Comparison between manual (performed by 4 researchers independently) and automated determination of layer boundaries. p-values from left to right: $p = 0.2722$, $p = 0.1512$, $p = 0.6850$, $p > 0.9999$, $p = 0.5720$, $p = 0.3005$, $p = 0.9134$, $p = 0.9248$. E) A mean deviation cut-off of 5 determines whether the nuclei follow a single (1 peak) or double (2 peak) Gaussian distribution. F) If the nuclei follow a single Gaussian distribution, the width of the peak vs. position of the peak determines organization. G) Mature monolayers display a two-step growth to the peak intensity reflected in the actin plot. G') The first derivative of the actin plot shows 2 peaks, with the apical peak being more prominent than the peak reflecting lateral surfaces. H) Intermediate layers have mono-peaked actin intensity plots therefore the derivative of Intermediate layers either has one peak (H') or the ratio of the right peak to the left peak is less than one. I) Manual counts of apically-positioned nuclei match with ALAn's counts. Manual counts performed blind by 4 researchers independently. X-axis categories represent different images. $p = 0.0011$, $p = 0.0708$, $p = 0.7120$, $p = 0.0764$. All statistics unpaired, two-tailed Student's t-test.

SUPPLEMENTAL FIGURE 2

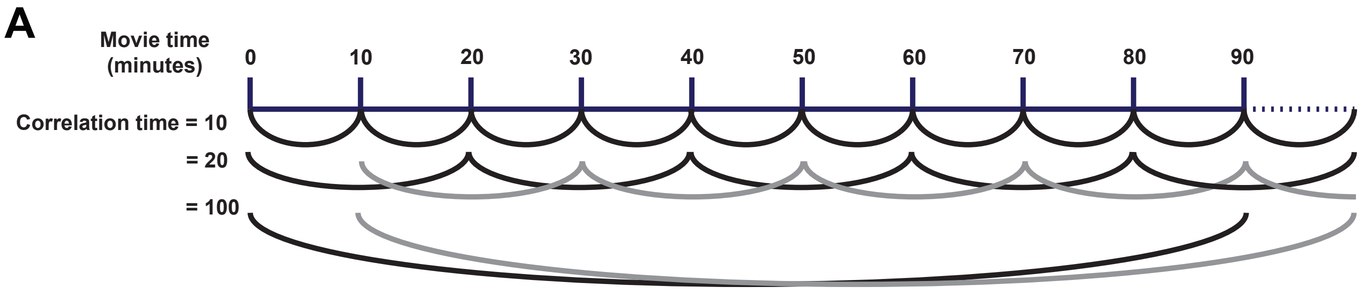

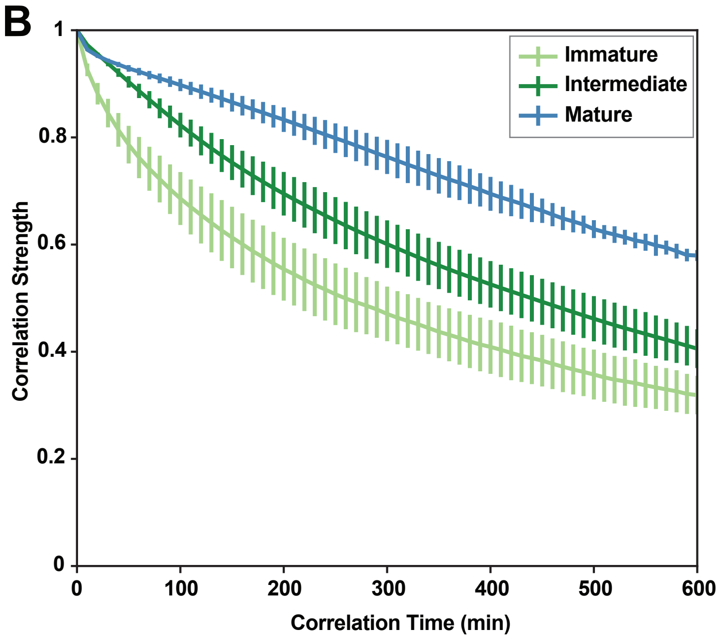

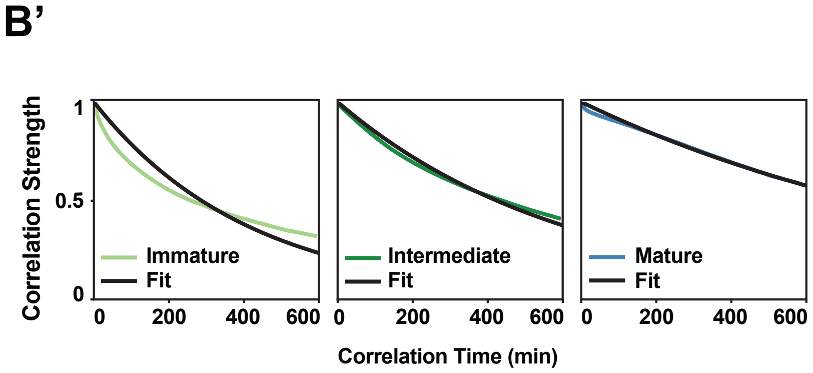

Supplemental Figure 2: Quantification of Layer Fluidity
A) Schematic representing how the quantification in B was performed. All movie frames separated by a given correlation time are compared and averaged to find the correlation strength. B) Supplemental Movies 1-3 are analyzed using the autocorrelation function and plotted together. The Mature layer decorrelates the slowest, given the blue curve remains at a higher correlation strength throughout the comparisons. B') The decay rate is measured for each movie by fitting a decaying exponential to each autocorrelation plot. The half-life for the layer types from left to right are 287, 424, and 754 minutes respectively.

SUPPLEMENTAL FIGURE 3

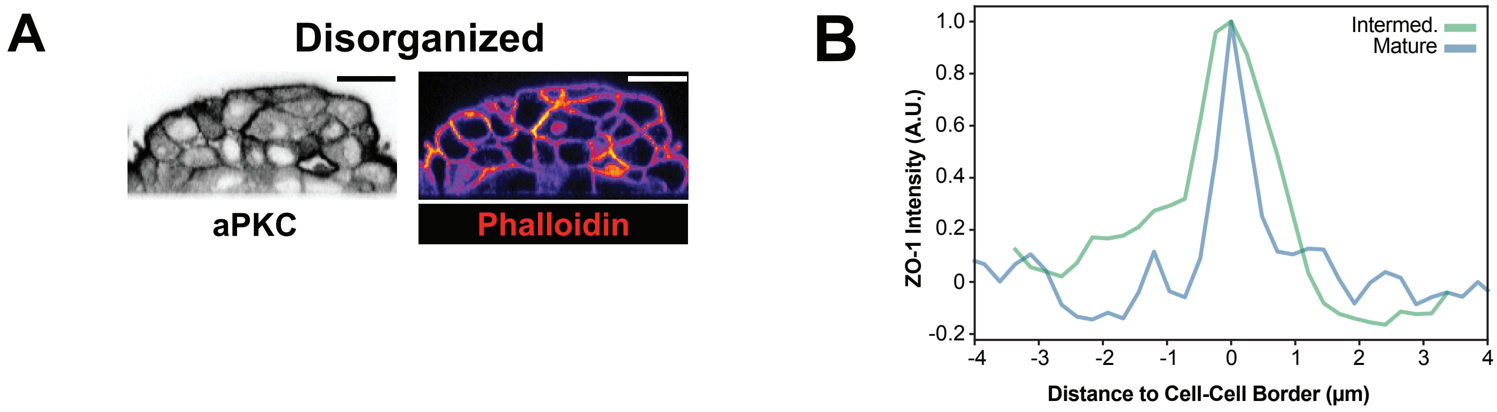

Supplemental Figure 3: Markers of polarity and adhesion in different layers.
A) aPKC is observed at all cell-cell borders in the disorganized regions. B) ZO-1 signal at the cell-cell border (position 0) is sharper in a Mature layer cell than an Immature layer cell.

Supplemental Figure 4

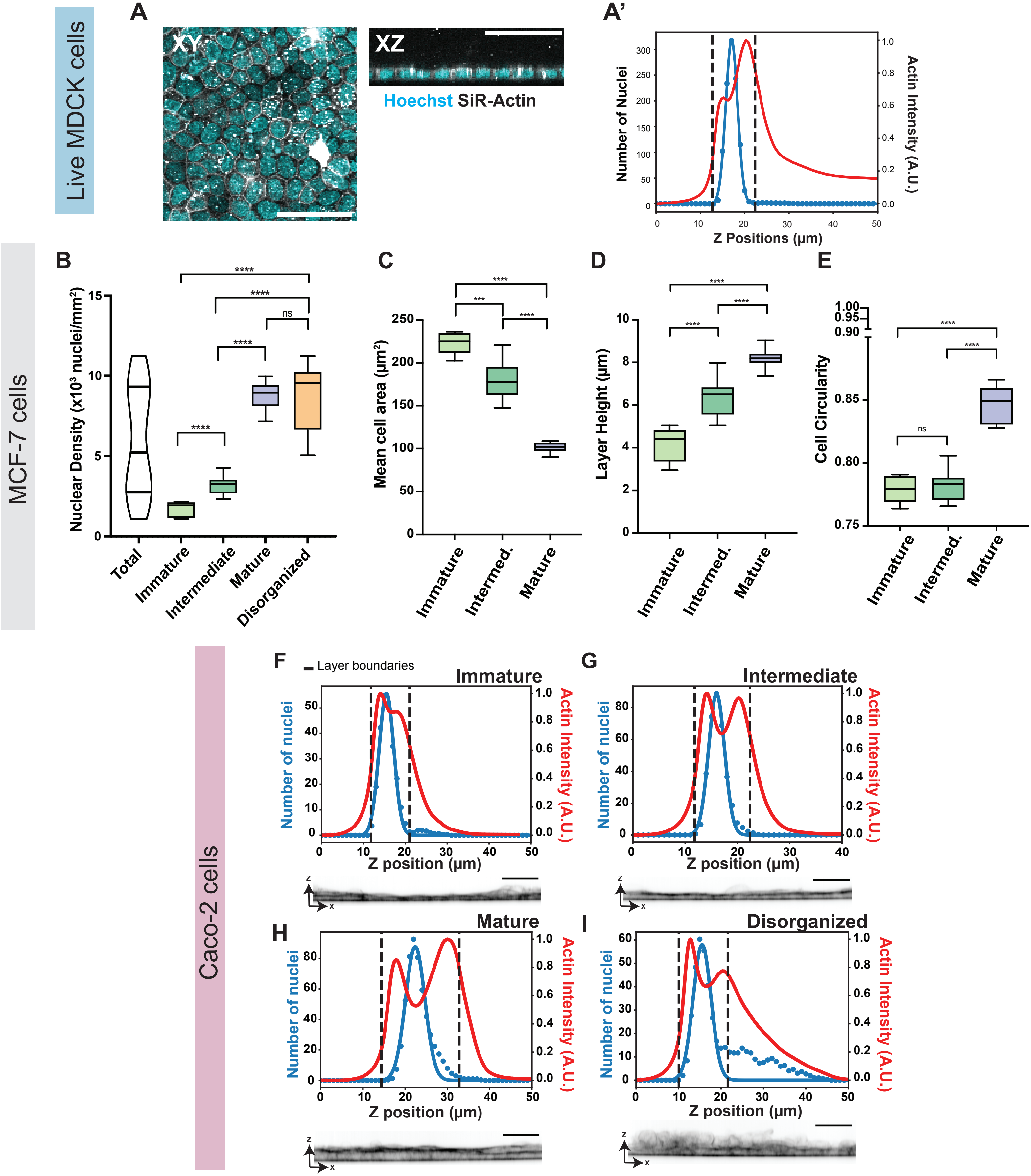

Supplemental Figure 4: Live MDCK, MCF-7 and Caco2 analysis with ALAn

A) Live imaging of MDCK cells stained with Hoechst and SiR-Actin produces promising plots for classiying live samples.B-E) Layer characteristics of MCF-7 cells are similar to those seen in MDCK cells. B) Densities of each layer type found in MCF-7 cell cultures cell area decreases as layers mature (C) while layer height (D) and cell circularity (E) increase as layers transition from Immature to Mature. Immature n = 5, Intermediate n = 20, Mature n = 10. E) . n = 7 Immature, 17 Intermediate, 10 Mature, 18 Disorganized. F-I) Example plots from Caco2 monolayer cultures representing Immature (F), Intermediate (G), Mature (H) and Disorganized (I) architectures. A prominent basal peak can be seen in all architectures.

Supplemental Figure 5

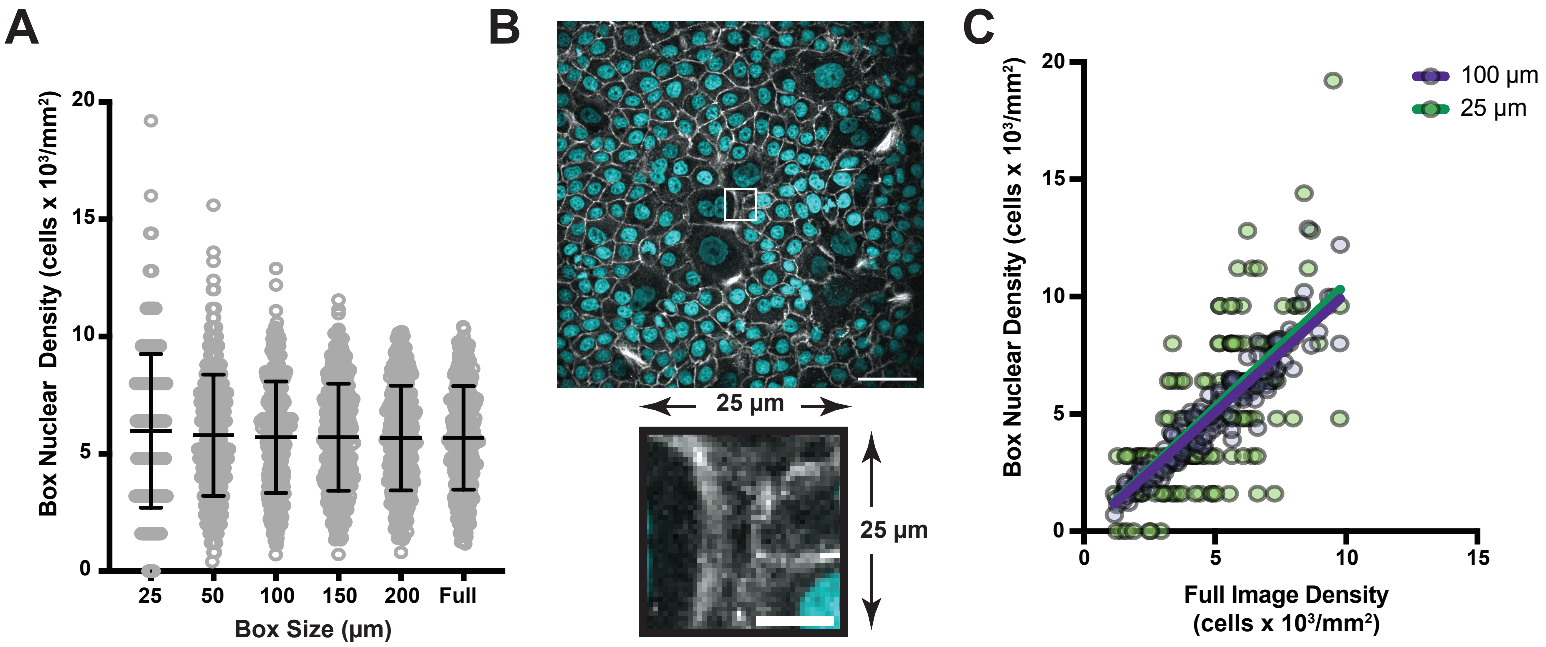

Supplemental Figure 5: Smaller regions accurately represent overall image density
A) Densities found within a range of box sizes from 25 µm x 25 µm up to the full image (~300 µm x 300 µm) show that while the spread of densities is greater in the smaller regions, the mean does not change meaning the density found in a 25 µm x 25 µm box accurately represents that of the full image. B) Representative image showing how some of the regions within the 25 µm x 25 µm boxes have a density of 0 cells x10^3/mm^2. C) Plotting the densities for 25 µm x 25 µm and 100 µm x 100 µm against the full image density shows correlations of ~1, again showing the densities in smaller regions are representative of the whole image.

SUPPLEMENTAL FIGURE 6

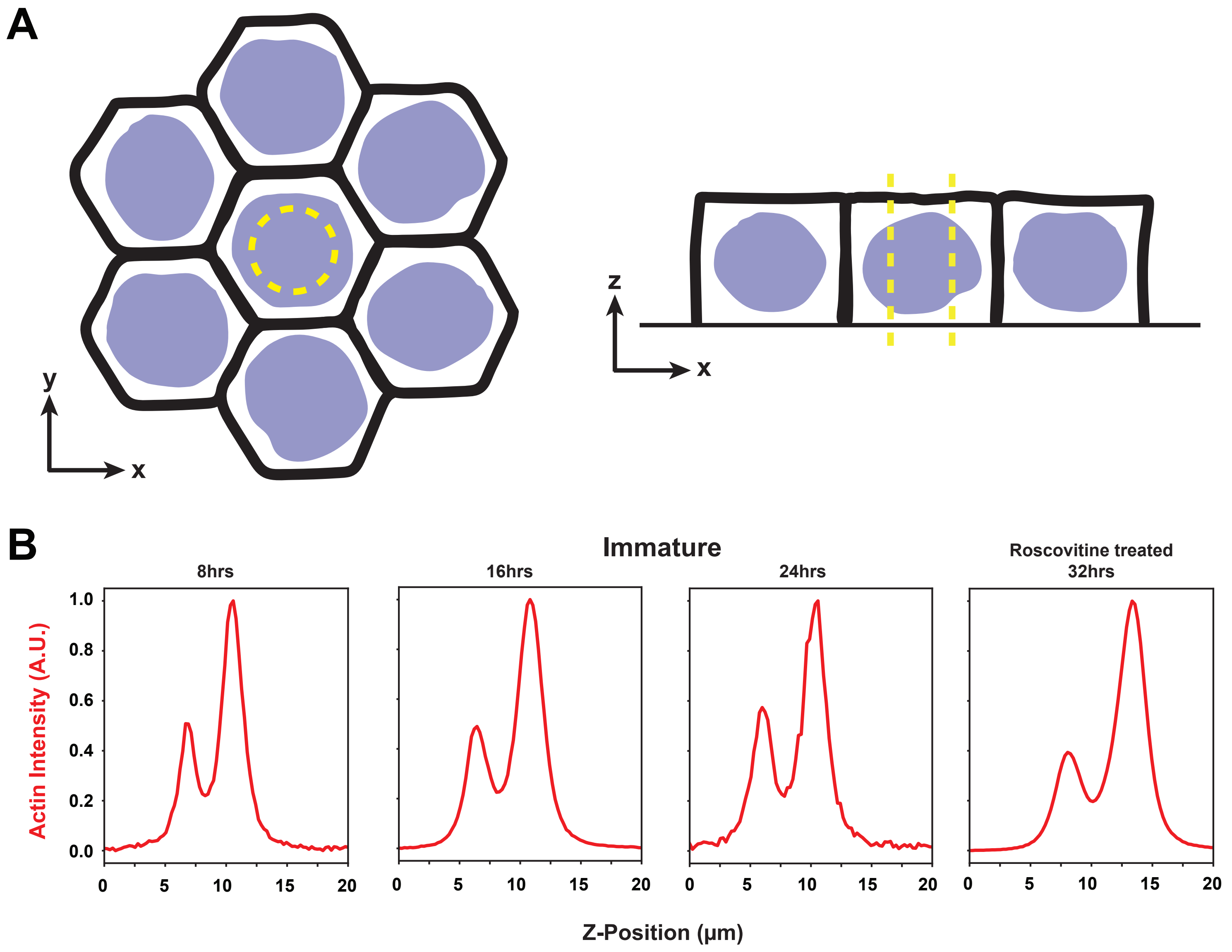

Supplemental Figure 6: Actin profiles of cells in Immature layers.
A) Single cell actin/aPKC plots were made by taking a circular section through center of the nucleus in the XY plane. The X and Y components are summed, resulting in an intensity vs. Z plot which captures apical and basal surfaces while avoiding lateral surfaces. B) Cortical actin distributions are independent of layer age. Asymmetry is the same in Immature cells at every timepoint examined, including in Roscovitine treated cells. Example plots are shown.

Supplemental Figure 7

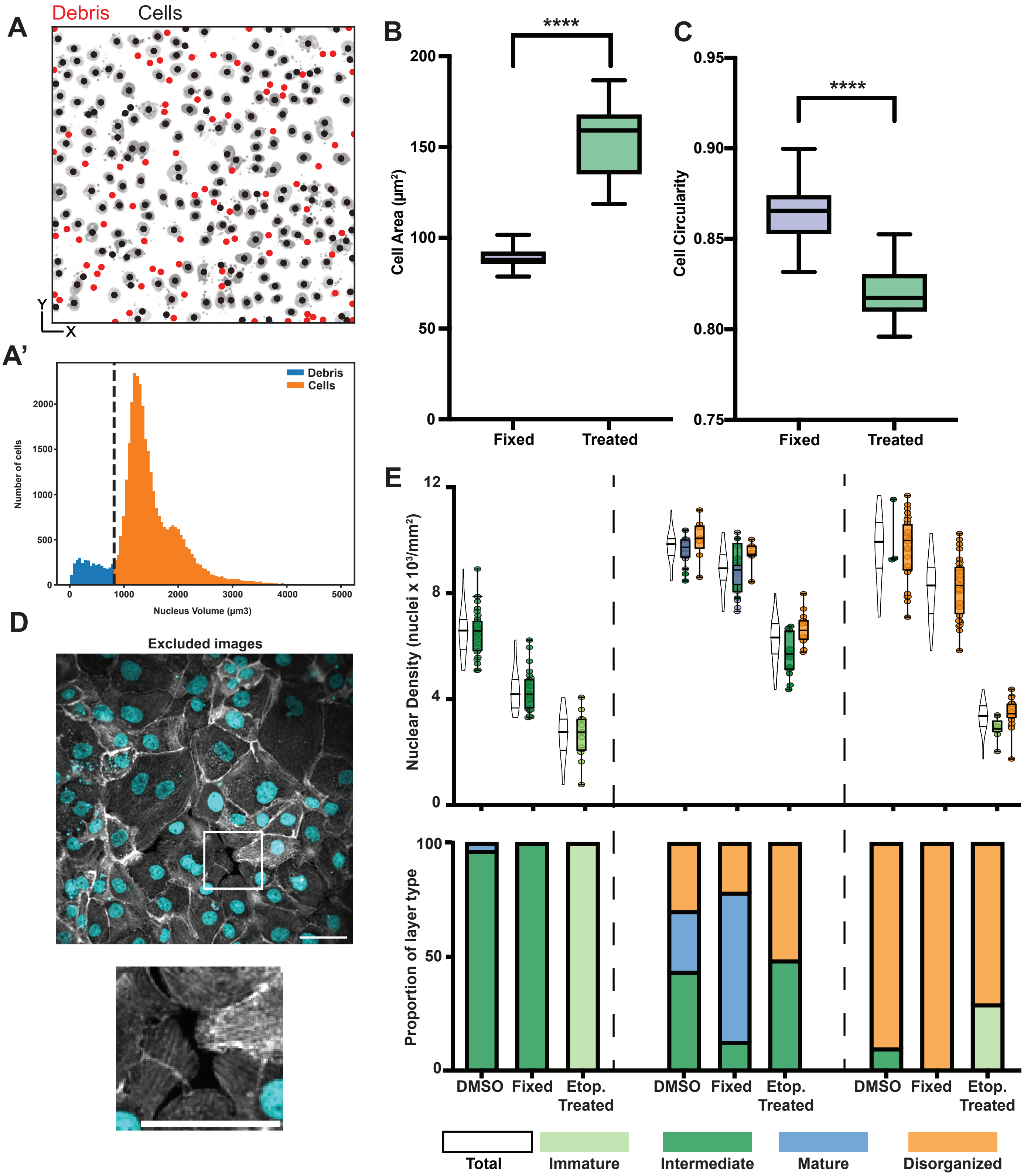

Supplemental Figure 7: Characteristics of Etoposide treated monolayers.
A) Pyknotic nuclei are seen throughout the etoposide treated monolayers which are included in the nuclear plot with a cut off of 1.5 standard deviations below the mean nuclear volume. A') Most of the pyknotic nuclei are excluded when a stricter cut off of 1 standard deviation below the mean is used. B, C) Characteristics of cells treated with etoposide are as expected. Cell area increases as monolayers reverse to Intermediate (B) while cell circularity decreases (C). D) Intermediate layers treated with etoposide results in some images with gaps between cells. These images were excluded from the analysis. E) Etoposide data from Figure 7 including DMSO controls.

SUPPLEMENTAL FIGURE 8

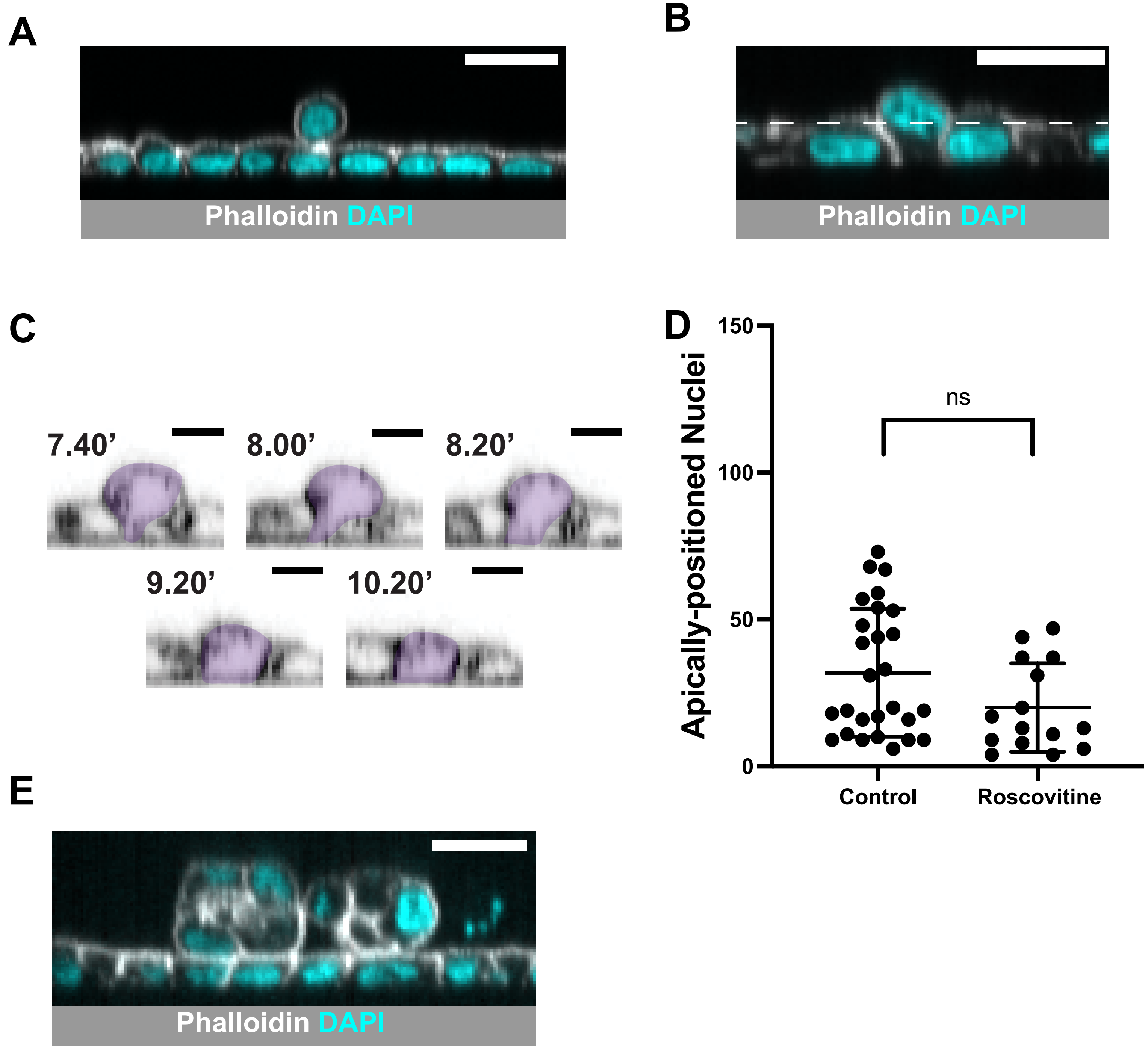

Supplemental Figure 8: Analysis of apically-positioned nuclei.
A) Representative image of a Mature monolayer with a cell on top of the monolayer. B) At 8 hours post seeding, rare (~2%) cells are attached to the substrate but not yet settled into the layer. The nuclei of these cells can be situated above the rest of the layer. Height of the layer (as determined by ALAn) is shown by the dashed line. C) Example of an apically-protruding cell (false colored) as it finishes settling past 8 hours post seeding. D) 200K MDCK cells were plated and allowed to develop for 24hrs, then treated with either DMSO or Roscovitine for a further 24hrs. Comparison between the two conditions shows no significant difference in the number of apically-positioned nuclei. Average densities: DMSO control = 9.22 x103 nuclei/mm2; Roscovitine treated = 7.04 x103 nuclei/mm2. p = 0.0683. E) Aggregates positioned on top of an organized monolayer. All statistics unpaired, two-tailed Student’s t-test.

SUPPLEMENTAL FIGURE 9

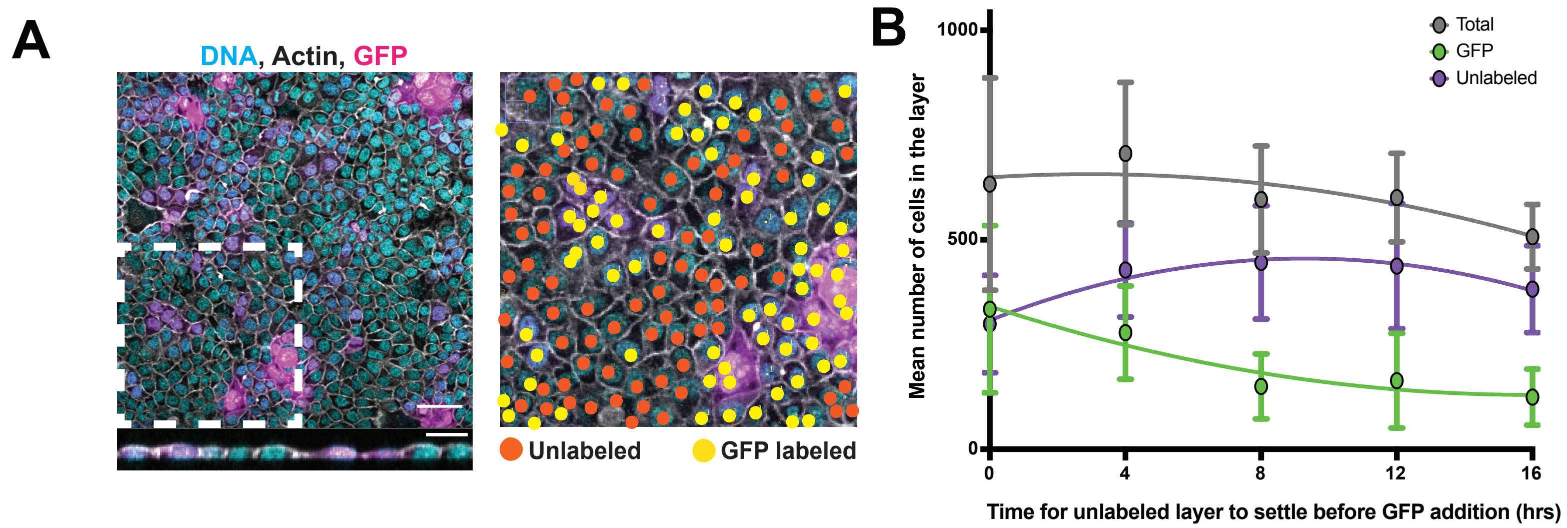

Supplemental Figure 9: Cell incorporation decreases as cells cover the substrate.
A) Representative image indicating how images were analyzed. Both unlabeled and GFP labeled cells on the substrate were manually counted using the Counter tool in FiJI. B) Cells seeded at a lower density (100K of each labeled and unlabeled) follow the same trend as cells seeded at a higher density.
