# Supplementary material for: A novel tool for the unbiased characterization of epithelial monolayer development in culture
Source: Mol Biol Cell. 2023 Mar 7;34(4):ar25. doi: 10.1091/mbc.E22-04-0121 (PMC10092640; doi:10.1091/mbc.E22-04-0121)
Supplement: Supplementary file 8 [file mbc-34-ar25-s002.pdf]

A novel tool for the unbiased characterization of epithelial monolayer development in culture

Nicole Dawney, Christian Cammarota, Qingyuan Jia, Alicia Shipley, Joseph Glichowski, Muskaan Vasandani, Tara Finegan, and Dan Bergstralh

Corresponding author(s): Dan Bergstralh, University of Rochester

Review Timeline:

Submission Date:	2022-04-11
Editorial Decision:	2022-05-19
Revision Received:	2022-12-09
Accepted:	2023-01-13

Editor-in-Chief: Matthew Welch

Transaction Report:

(Note: With the exception of the correction of typographical or spelling errors that could be a source of ambiguity, letters and reports are not edited. The original formatting of letters and referee reports may not be reflected in this compilation.)

RE: Manuscript #E22-04-0121
TITLE: "An unbiased characterization of epithelial monolayer development in culture"

Dear Dan,

Thank you for sending us your manuscript for consideration at MBoC. It has now been seen by two reviewers, whose comments follow below.

Unfortunately, the news is not good. Both reviewers identify the potential for your new tool to be a useful resource for the field, but feel that it has not been applied and validated to the extent that would be necessary. Similarly, the analysis of tissue fluidity is not pursued to a degree that would yield significant new insight.

Overall, very extensive development and new experimentation would be required for your manuscript to appeal to the general cell biological audience of MBoC. I think that this is beyond the reasonable scope of a revision and so must decline to consider it further for MBoC.

I'm sorry that our decision is negative in this instance, and hope that our reviewers' comments are useful for you.

Best wishes,

Alpha

Alpha Yap
Monitoring Editor
Molecular Biology of the Cell

Reviewer #1 (Remarks to the Author):

Epithelial tissue is known to be inhomogeneous. In this manuscript, Dawney, Cammarota, and the team developed an image analysis tool (ALAn) and qualitatively categorized the different architectures of MDCK tissue. Four different architectures are correlated with cell density. Moreover, by using live imaging, the authors characterized how cells that are not originally attached to the substrate are integrated into a tissue.
It is not clear to me, whether the authors are mainly intending to 1) introduce and demonstrate a new tool, or 2) use this new tool and find something biologically novel. As it stands, the manuscript is somewhere in the middle. As a consequence, the manuscript is somewhat immature in either intention at this stage.

Major comments

1. If the intention is to 1) introduce and demonstrate a new tool:
1-1. The experiments tracing the dynamics of cells using live imaging are not taking advantage of ALAn. As a consequence, the data shown in Fig. 6 and 7 looks somewhat disconnected from Fig. 1-5.
1-2. The fluorescent marker used in these live imaging (for instance, Fig. 6D and 7B), are not actin, and thus they are not compatible with ALAn.
1-3. It would be valuable if the authors demonstrate that ALAn can be used for the 4D movie (3D + time). MDCK with actin-GFP (or GFP tagged to actin-associated protein) and Hoechst staining should be helpful.
1-4. It would be powerful if the authors extend ALAn so that the algorism map (and color codes) the different architectures within a tissue. For instance, the pink square with numbers shown in Fig.1B middle can be replaced by a color-coded square (the colors correspond to the different architectures shown in Fig. 1D).
1-5. It would be valuable to the community if the suggestions 1-3 and 1-4 are both incorporated into ALAn. This updated tool could highlight "the importance of accurately determining monolayer architecture when using cultured cells to study epithelial biology" even stronger.
1-6. The description of ALAn can be expanded, especially the section "ALAn further subcategorizes these into... (line 122)".

2. If the intention is to 2) use this new tool and find something biologically novel:
2-1. The data are purely observations without mechanism, and most of them are known cell-density-dependent phenomena. Thus, I would say this study suffers from a lack of biological novelty.
2-2. One of the interesting ideas the authors put forward is "tissue fluidity in the apical basal plane", "fluidity transition", and

"jamming transition", which are all related. I'm not sure the data shown in this study can be considered as jamming/fluidity transition. The data including the summary figure in Fig. 7c show that 1) the cells on top of the other cells can rearrange and adhere to the substrate if there are open spaces (low density), but 2) the most cells can't be incorporated into the tissue if there is no space (high density). To me, this is simply a density problem, as the authors concluded, and not fluidity/jamming transition.
2-3. To demonstrate these phenomena are fluidity/jamming transitions, more characterizations need to be done. For instance, 1) how the cell-cell adhesion, including the dynamics of E-cadherin at cell-cell junction, is altered, and 2) how cell motility is changed (Is cell migration toward the substrate active or passive?). To further support these observations, some perturbations, such as using drugs to alter the dynamics of cell-cell adhesion or cell motility, and ask whether the fluidity/jamming transition is altered.
2-4. Is the disorganized tissue shown in Fig. 2D formed with the same mechanism shown in Fig. 7c?

3. My feeling is Comment 1 is doable in comparison to Comment 2.

Minor comments

4. I would suggest changing the following wording.
Line 257: their ability to reorganize in depth
Line 273: The cells ... reorganize downward
Line 337: mitotic cell daughter displacement

5. Line 165: I can't see the following description in the figure cited. "This enrichment is strongest in Mature layers followed by Intermediate, but less obvious in Immature layers (Fig. 3D-F column 2)."

6. Fig. 3D-F columns 3 and 4: Additional quantifications would be helpful.

7. There are no descriptions of the data shown in Fig. 4A' and B'.

8. Fig. S1F-F": How do you segregate the green dots and orange dots, and fit them separately?

Reviewer #2 (Remarks to the Author):

The manuscript by Dawney and Cammarota et al, reports on the characterization and precise measurements of epithelial monolayer architecture development in culture using an automated (imaging) layer analysis (ALAn) method.
Using this tool, they report that epithelial architecture correlates with cell density and that tissue fluidity in the apical-basal axis is governed by substrate availability.
Although the aspects covered in this work could be of interest for MBoC broad audience, I have two main concerns that prevents my recommendation for publication in MBoC.

1. The author's reports on a new tool to measure epithelial monolayer architecture. This is based primarily on the z-positioning of nuclei, the circumferential actin belt and basal F-actin, determined in monolayers stained with phalloidin (F-actin) and Hoechst (Nuclei).

Several methods exist to measure monolayer architecture, with most of them focusing on the "top-view" architecture. Yet, less attention has been given to measuring basal-axial architecture, which is a novel aspect of this work. Yet, most of the time, image analysis methods for the analysis of epithelia work from good to excellent and present significant variability across the type of monolayer tissue to be analysed, quality of images, etc.

From my point of view, what is missing in this manuscript, is that if this is a new method that can be broadly adopted by the research community, it needs more data that further validate the robustness of this approach. It would be desirable, that the method is applied to monolayers for other cell lines (e.g. MCF-7/10, Caco-2, A-431) as well as epithelial tissues derived from animal models (e.g. tissue sections). Also, in using cell lines,

2. Using ALAn, the authors report that epithelial architecture correlates with cell density and that tissue fluidity in the apical-basal axis is governed by substrate availability.
i. Figure 3A. QUantitatitive measurements of T1 transitions and individual cell movements within the monolayer in monolayers of different architectures is needed to support the conclusion that epithelial architecture correlates with tissue fluidity
ii. IF substrate availability is critical for incorporation of new cells, would then generating substrate space "reverse" the disorganized phenotype to a more mature phenotype. This could be achieved by stretching the substrate (e.g. using stretchable substrates) or by inducing stochastic cell death and cell extrusions from the monolayer (i.e. by treatment by Etoposide, Rossenblatt, JCB, 2001).

We provide below a point-by-point response detailing how our new manuscript addresses the Reviewer's major comments from our previous submission. (RE: Manuscript #E22-04-0121, TITLE: "An unbiased characterization of epithelial monolayer development in culture", comments received May 19th 2022). We have not addressed Reviewer 1's minor comments because they have been resolved in our complete reworking of the paper.

Reviewer #1 (Remarks to the Author):

Epithelial tissue is known to be inhomogeneous. In this manuscript, Dawney, Cammarota, and the team developed an image analysis tool (ALAn) and qualitatively categorized the different architectures of MDCK tissue. Four different architectures are correlated with cell density. Moreover, by using live imaging, the authors characterized how cells that are not originally attached to the substrate are integrated into a tissue.
It is not clear to me, whether the authors are mainly intending to 1) introduce and demonstrate a new tool, or 2) use this new tool and find something biologically novel. As it stands, the manuscript is somewhere in the middle. As a consequence, the manuscript is somewhat immature in either intention at this stage.

- We have substantially revised our new manuscript to emphasize our paper as an introduction of a new tool. We consider the manuscript and our analysis tool to be a resource for the community to classify 3D layer architecture in cultured cells. We have retained the quantitative analysis of the biological and material properties of the layer architectures tool. We present some perturbation experiments for which ALAn has been used to understand the mechanisms underlying architecture changes.

Major comments

1. If the intention is to 1) introduce and demonstrate a new tool:
1-1. The experiments tracing the dynamics of cells using live imaging are not taking advantage of ALAn. As a consequence, the data shown in Fig. 6 and 7 looks somewhat disconnected from Fig. 1-5.
1-2. The fluorescent marker used in these live imaging (for instance, Fig. 6D and 7B), are not actin, and thus they are not compatible with ALAn.
1-3. It would be valuable if the authors demonstrate that ALAn can be used for the 4D movie (3D + time). MDCK with actin-GFP (or GFP tagged to actin-associated protein) and Hoechst staining should be helpful.

- We agreed that it would be exciting if ALAn can be used for the characterization of layers in 4D. ALAn requires homogenous actin signal across the layer. The tool is based on averaging an area of cells together to visualize a distribution of actin along the apical-basal axis, and heterogeneous expression invalidates this approach. We tried several visualization options to test the applicability of ALAn on live samples. Layers plated at different seeding densities were left for 24 hours and imaged live using Hoechst or H2B-mCherry to segment nuclei and SiR-actin and LifeAct-GFP to visualize actin, and

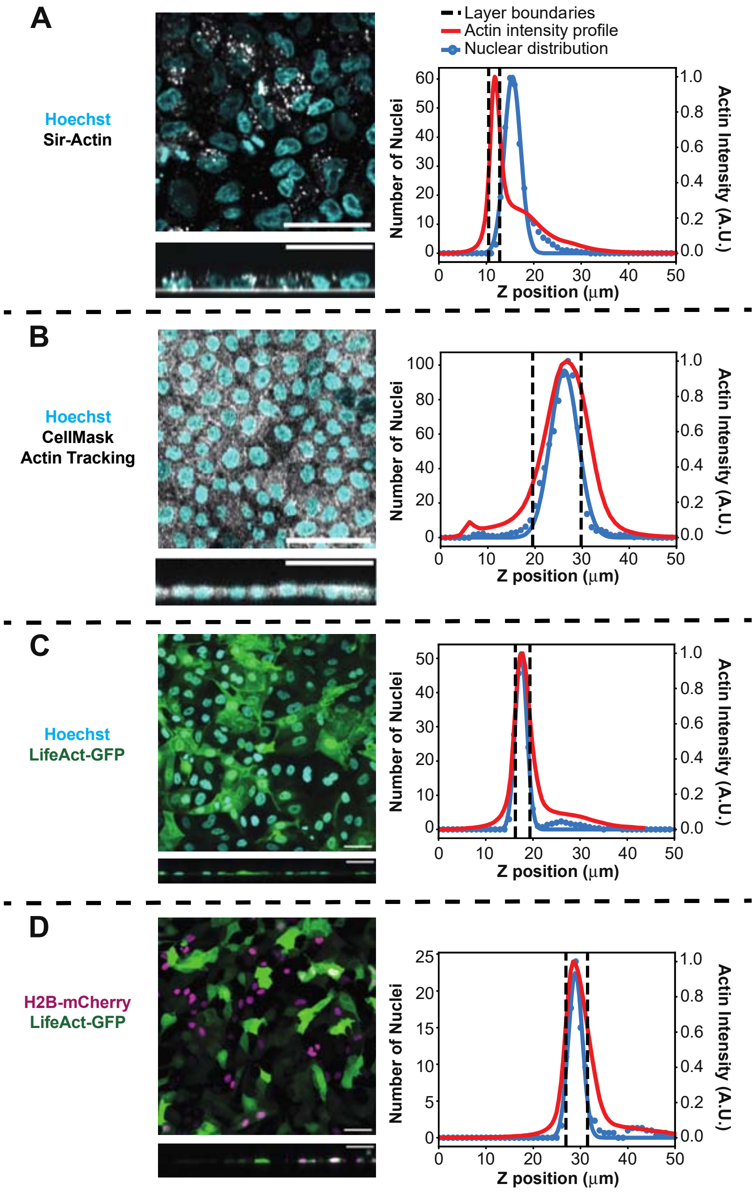

Figure: Inconsistencies with vital dyes along with heterogeneity make use of ALAn on live samples difficult.
A-D) Example plots produced from different live stains/markers in order to test ALAn on live samples. A) SiR-Actin often produced a prominent basal peak and no lateral staining, particularly at lower densities. B) While lateral borders are able to be seen in the live imaging with CellMask Actin Tracking, the level of background was too high for ALAn to accurately determine layer architecture. C, D) Expression of LifeAct-GFP (C) and LifeAct-GFP with H2B-mCherry (D) are too heterogenous for use with ALAn.

CellMask to visualize junctions (Figure, above). We found SiR-actin and LifeAct-GFP signal to be unpredictable in our hands and thus cell borders were not always detectable in our samples, particularly at lower densities. The SiR-actin profile generally reflects the layer architecture profiles distinguished from Phalloidin staining but background signal above the layer changes the righthand side of the profile (manuscript new Figure 4G,G').

We expect that with cell line that expresses homogenous LifeAct-GFP could be used. In conclusion, although we struggled with heterogeneities in markers, we have in principle shown that ALAn can be used to characterize layer architecture in live data, and have added this to the manuscript. Due to our difficulties in obtaining high-quality movie data, we have not extended the ALAn code such that it can automatically analyze 4D movies. However, high quality images from 4D movies can be readily input into ALAn to analyze architecture changes across time.

1-4. It would be powerful if the authors extend ALAn so that the algorism map (and color codes) the different architectures within a tissue. For instance, the pink square with numbers shown in Fig.1B middle can be replaced by a color-coded square (the colors correspond to the different architectures shown in Fig. 1D).
1-5. It would be valuable to the community if the suggestions 1-3 and 1-4 are both incorporated into ALAn. This updated tool could highlight "the importance of accurately determining monolayer architecture when using cultured cells to study epithelial biology" even stronger.

- We understood the reviewer's suggestion to mean that we should color code the entire 1cm x 1cm well based on ALAn-generated architecture. However, this is not feasible due to huge amount of data this would generate (1000 4G stacks = 4TB per one well). For each well, we image only 1% of the entire surface area, albeit in representative locations. We were however inspired by the reviewer's suggestion to enhance the tool. We ran smaller regions of our images through ALAn and show that small areas of an image correlate with the overall architecture of the image. We have added color-coded submaps to represent the layer architectures across different areas to illustrate this result (new manuscript Figure 5E.) We can confidently use ALAn on images as small as ~100μm across. We show that even on this scale images can vary. We have presented these new results in our new manuscript in Figures 5E and S5.

2. If the intention is to 2) use this new tool and find something biologically novel:
2-1. The data are purely observations without mechanism, and most of them are known cell-density-dependent phenomena. Thus, I would say this study suffers from a lack of biological novelty.

- We have reframed our paper to be tool-oriented, but we do want to provide a response to these reviewer comments. Our data shows that the process of developing into a Mature monolayer is not solely density dependent (if we increase the seeding density to that of a Mature layer we get Disorganization as a result). We further explain why and how this Disorganization arises, thus we disagree with the comment of a 'lack of biological novelty'. Additionally a major focus of the paper is the ability for cells to reorganize out of the traditional tissue plane into the common planar organization for MDCK cells. We hope that in rewriting and reframing our paper we have made explicit how our work reveals that 3D architecture leads to differences in the biological and material properties of epithelial layers.

2-2. One of the interesting ideas the authors put forward is "tissue fluidity in the apical basal plane", "fluidity transition", and "jamming transition", which are all related. I'm not sure the data shown in this study can be considered as jamming/fluidity transition. The data including the summary figure in Fig. 7c show that 1) the cells on top of the other cells can rearrange and adhere to the substrate if there are open spaces (low density), but 2) the most cells can't be incorporated into the tissue if there is no space (high density). To me, this is simply a density problem, as the authors concluded, and not fluidity/jamming transition.

2-3. To demonstrate these phenomena are fluidity/jamming transitions, more characterizations need to be done. For instance, 1) how the cell-cell adhesion, including the dynamics of E-cadherin at cell-cell junction, is altered, and 2) how cell motility is changed (Is cell migration toward the substrate active or passive?). To further support these observations, some perturbations, such as using drugs to alter the dynamics of cell-cell adhesion or cell motility, and ask whether the fluidity/jamming transition is altered.
2-4. Is the disorganized tissue shown in Fig. 2D formed with the same mechanism shown in Fig. 7c?

- Density changes are a well-established and accepted mechanism to explain tissue jamming/fluidity transitions in the plane of a tissue (reviewed by Elizabeth Lawson-Keister, M. Lisa Manning "Jamming and arrest of cell motion in biological tissues" (2021) Current Opinion in Cell Biology, Volume 72 146-155) and hence we disagree with the reviewer's comment that the rheological change (change in the fluidity of the tissue) we note between architectures is not a fluidity/jamming transition.

Reviewer #2 (Remarks to the Author):

The manuscript by Dawney and Cammarota et al, reports on the characterization and precise measurements of epithelial monolayer architecture development in culture using an automated (imaging) layer analysis (ALAn) method.
Using this tool, they report that epithelial architecture correlates with cell density and that tissue fluidity in the apical-basal axis is governed by substrate availability.
Although the aspects covered in this work could be of interest for MBoC broad audience, I have two main concerns that prevents my recommendation for publication in MBoC.

1. The author's reports on a new tool to measure epithelial monolayer architecture. This is based primarily on the z-positioning of nuclei, the circumferential actin belt and basal F-actin, determined in monolayers stained with phalloidin (F-actin) and Hoechst (Nuclei).

Several methods exist to measure monolayer architecture, with most of them focusing on the "top-view" architecture. Yet, less attention has been given to measuring basal-axial architecture, which is a novel aspect of this work. Yet, most of the time, image analysis methods for the analysis of epithelia work from good to excellent and present significant variability across the

type of monolayer tissue to be analysed, quality of images, etc.

From my point of view, what is missing in this manuscript, is that if this is a new method that can be broadly adopted by the research community, it needs more data that further validate the robustness of this approach. It would be desirable, that the method is applied to monolayers for other cell lines (e.g. MCF-7/10, Caco-2, A-431) as well as epithelial tissues derived from animal models (e.g. tissue sections).

- Our new manuscript now details how ALAn can be used to analyze and characterize the architecture of MCF-7 and Caco-2 cell lines (new manuscript Figures 5 and S4). We believe that tissue sections are outside of the scope of our work, as ALAn has been developed to analyze tissues imaged in 3D using confocal microscopy.

2. Using ALAn, the authors report that epithelial architecture correlates with cell density and that tissue fluidity in the apical-basal axis is governed by substrate availability.
i. Figure 3A. Quantitative measurements of T1 transitions and individual cell movements within the monolayer in monolayers of different architectures is needed to support the conclusion that epithelial architecture correlates with tissue fluidity

- We performed the quantitative analysis of T1 rate that the reviewer suggested and have added this analysis and result to our new manuscript (Figure 4C). We find that Immature architectures undergo T1 transitions at a rate of ~10 times greater than the intermediate and mature counterparts. We also used second technique to test how quickly images ‘decorrelate’, essentially quantifying how quickly the cells are changing (shape, position, organization etc.). The correlation analysis compares two images on a -1 to 1 scale where -1 means the images are completely anticorrelated (inverse), 0 uncorrelated, and 1 completely correlated (identical). The images we compare are frames of a time course separated by a correlation time This analysis in presented in our new manuscript in Supplemental Figure 2. Furthermore, each of these autocorrelation functions were fit to a decaying exponential to determine a characteristic decay time for each of the layer types. The half-life for the Immature movie in Supplemental Movie 1 is 287 minutes, while the half life for the Mature movie in Supplemental Movie 3 is almost three times longer at 754 minutes.

ii. IF substrate availability is critical for incorporation of new cells, would then generating substrate space "reverse" the disorganized phenotype to a more mature phenotype. This could be achieved by stretching the substrate (e.g. using stretchable substrates) or by inducing stochastic cell death and cell extrusions from the monolayer (i.e. by treatment by Etoposide, Rossenblatt, JCB, 2001).

- This was a terrific idea for an experiment. We worked on both the proposed idea as well as an additional set of experiments to further probe the findings that architectures transition as layers densify. We find that de-densifying layers using

Etopside can lead to a reversion in architectures from intermediate to immature layer types. Furthermore, we found a similar trend for the reversion of mature to intermediate layers. With respect to disorganization, we found that induced cell death can lead to lower density disorganized layers, but we see a population of layers which revert from a disorganized state to an organized state. These results are presented in our new manuscript in Figure 8.

RE: Manuscript #E22-04-0121R-A
TITLE: "A novel tool for the unbiased characterization of epithelial monolayer development in culture"

Dear Dan,

Thank you for sending us your revised manuscript. Apologies for the delay in reviewing it - the holidays! Happily, as you can see, both reviewers support its publication. So, I am pleased to accept it for MBoC.

You will note one minor typo that was noted by Reviewer 2. But I think that this can be caught in the proofs.

Best,

Alpha

Alpha Yap
Monitoring Editor
Molecular Biology of the Cell

Dear Dr. Bergstralh:

Congratulations on the acceptance of your manuscript.

A PDF of your manuscript will be published on MBoC in Press, an early release version of the journal, within 10 days. The date your manuscript appears at www.molbiolcell.org/toc/mboc/0/0 is the official publication date. Your manuscript will also be scheduled for publication in the next available issue of MBoC.

Within approximately four weeks you will receive a PDF page proof of your article.

Would you like to see an image related to your accepted manuscript on the cover of MBoC? Please contact the MBoC Editorial Office at mboc@ascb.org to learn how to submit an image.

Authors of Articles and Brief Communications are encouraged to create a short video abstract to accompany their article when it is published. These video abstracts, known as Science Sketches, are up to 2 minutes long and will be published on YouTube and then embedded in the article abstract. Science Sketch Editors on the MBoC Editorial Board will provide guidance as you prepare your video. Information about how to prepare and submit a video abstract is available at www.molbiolcell.org/science-sketches. Please contact mboc@ascb.org if you are interested in creating a Science Sketch.

We are pleased that you chose to publish your work in MBoC.

Sincerely,

Eric Baker
Journal Production Manager
MBoC Editorial Office
mbc@ascb.org

Reviewer #1 (Remarks to the Author):

In this revised manuscript, the authors have adequately addressed all of the questions and requests raised during the review process. The manuscript has undergone significant improvement, and the inclusion of new data and diagrams in Figures 2, 5, and 8 is commendable. I endorse this manuscript for publication.

Reviewer #2 (Remarks to the Author):

I authors have addressed most of my comments satisfactorily. This included new experiments showing how de-densification alters 3D monolayer organization. This provided experimental evidence of a model of race to the bottom for cells to be able to form organized mature monolayers.
Regarding the broad applicability of ALAN for the analysis of epithelial tissues, I understand its limitations in tissue sections. This could be the subject of a follow-up manuscript, which will be very interesting.
A little coment, Figure 5E was cited as Figure 5F in the text.
